# PIM1 Attenuates Innate Immunity to Foster Coronavirus Replication through Ubiquitin Ligase β‐TrCP‐Mediated IFNAR1 Degradation

**DOI:** 10.1002/advs.202503487

**Published:** 2025-07-06

**Authors:** Qianya Wan, Lin Zhu, Cien Chen, Li Zhong, Houying Leung, Wei Li, Chang Xu, Xi Yao, Huan Hu, Mandi Wu, Yuxin Hou, Hin Chu, Yiran Wang, Sheng Chen, Mingyu Pan, Zongwei Cai, Ming‐liang He

**Affiliations:** ^1^ Department of Biomedical Sciences and Tung Biomedical Sciences Center City University of Hong Kong Hong Kong 999077 China; ^2^ State Key Laboratory of Environmental and Biological Analysis Department of Chemistry, Hong Kong Baptist University Hong Kong 999077 China; ^3^ CityU Shenzhen Research Institute Nanshan Shenzhen 518057 China; ^4^ Weihai Municipal Hospital Cheeloo College of Medicine Shandong University Shandong 264200 China; ^5^ College of Medical Laboratory Dalian Medical University Dalian Liaoning 116044 China; ^6^ State Key Laboratory of Emerging Infectious Diseases Department of Microbiology Li Ka Shing Faculty of Medicine The University of Hong Kong Hong Kong 999077 China; ^7^ School of Pharmacy Nanjing Medical University Nanjing 211166 China

**Keywords:** β‐TrCP1, β‐coronavirus, IFNAR1, PIM1, ubiquitination

## Abstract

Virus infection stimulates proto‐oncoprotein PIM1 kinase expression, but its importance and the biological functions of this process are poorly understood. Herein, PIM1 promotes IFNAR1 degradation to attenuate cellular innate immunity during human coronavirus HCoV‐OC43 infection. During virus replication, the double‐stranded viral RNA and some viral proteins upregulate PIM1 expression, which phosphorylates E3 ubiquitin ligase β‐TrCP1 at Serine 82. The pS82‐β‐TrCP1 then forms a complex with S535/S539‐phosphorylated interferon receptor IFNAR1 (pS535/539‐IFNAR1), leading to IFNAR1 ubiquitination and degradation. Both pan‐inhibitors (CX‐6528, SGI‐1776, AZD‐1208) and a specific inhibitor of PIM1 kinase (PIM1 inhibitor 2) effectively block this process and potently inhibit viral replication. This studies demonstrate a novel strategy that viruses use to disrupt cellular innate immunity, suggesting a potential therapeutic target for further anti‐virus drug development.

## Graphical abstract

Hijacking Host Kinase PIM1: A β‐Coronavirus (OC43) strategy to degrade IFNAR1 and evade antiviral immunity. Human β‐coronaviruses (OC43) exploit the proto‐oncoprotein PIM1 kinase to sabotage innate immunity. Viral RNA/proteins upregulate PIM1, which phosphorylates E3 ligase β‐TrCP1. This primes β‐TrCP1 to bind p‐IFNAR1 (pS535/S539), triggering its ubiquitination and degradation. Impairing this interferon receptor, viruses enhance replication. Crucially, PIM1 inhibitors block this axis and suppress viral propagation, revealing PIM1 as a druggable target for coronavirus therapeutics. TOC graphic is created with BioRender.com and is reproduced under an academic subscription license (2025).

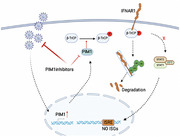

## 1 Introduction

Innate immunity, comprised of interferon production and downstream interferon response, serves as the primary defense against pathogen infection in vertebrates. Type I interferons (IFN) initiates interferon response by binding to the IFNα/β receptor subunit 1 (IFNAR1) and IFNAR2, and subsequently induces the formation of the STAT1–STAT2–IRF9 transcriptional complex (ISGF3). ISGF3 translocates into the nucleus and binds to the IFN‐stimulated response element (ISRE), inducing the expression of antiviral interferon‐stimulated genes (ISGs).^[^ ^1^, ^2^ ^]^

The sensitivity of cells to type I IFN relies on the density of IFNAR receptors on the cell surface.^[^ ^3^ ^]^ The level of IFNAR1 is governed by lysosomal degradation as a negative feedback mechanism to mitigate cytotoxic effects associated with ISG expression. This degradation involves phosphorylation‐dependent ubiquitination by the beta‐transducing repeat containing E3 ubiquitin protein ligase (β‐TrCP1).^[^ ^4^, ^5^ ^]^ Ubiquitination of IFNAR1 exposes its internalization motif, promoting the internalization and lysosomal degradation process.^[^ ^5^ ^]^ Viruses, including enterovirus A71,^[^ ^6^, ^7^ ^]^ influenza A virus (IAV),^[^ ^8^, ^9^ ^]^ Flavivirus,^[^ ^10^ ^]^ and hepatitis B virus (HBV),^[^ ^11^ ^]^ harness this mechanism to reduce IFNAR1 levels and evade host cell defense. Coronavirus also utilizes diverse strategies to escape innate immunity,^[^ ^12^ ^]^ while the serum IFNAR1 abundance inversely correlated with COVID‐19 severity.^[^ ^13^ ^]^ However, the exact mechanisms remain unclear.

Proviral integration site for Moloney murine leukemia virus‐1 (PIM1), a serine/threonine kinase, is a proto‐oncogene implicated in tumorigenesis, cell cycle, proliferation, cell growth, and apoptosis.^[^ ^14^, ^15^ ^]^ Recently, PIM1 was found to maintain proper activation of guanylate binding protein 1 (GBP1), facilitating pathogen clearance.^[^ ^16^ ^]^ PIM1 expression is upregulated during virus infection,^[^ ^17^, ^18^, ^19^ ^]^ decreasing interferon production and aiding virus evasion from host defence.^[^ ^20^, ^21^ ^]^ Infections by human β‐coronaviruses, such as HCoV‐OC43 (OC43), MERS‐CoV, SARS‐CoV, and SARS‐CoV‐2, can cause severe acute respiratory syndrome and other disorders, even to death.^[^ ^22^ ^]^ The role of PIM1 in modulating interferon signaling during human β‐coronavirus infection has not been reported. In this study, we observed an increase in PIM1 expression during human β‐coronavirus infection and dissected the underlying mechanism of how PIM1 enhanced virus infection.

## 2 Results

### 2.1 *β*‐Coronavirus Infection Stimulates PIM1 Expression

**Figure 1:**
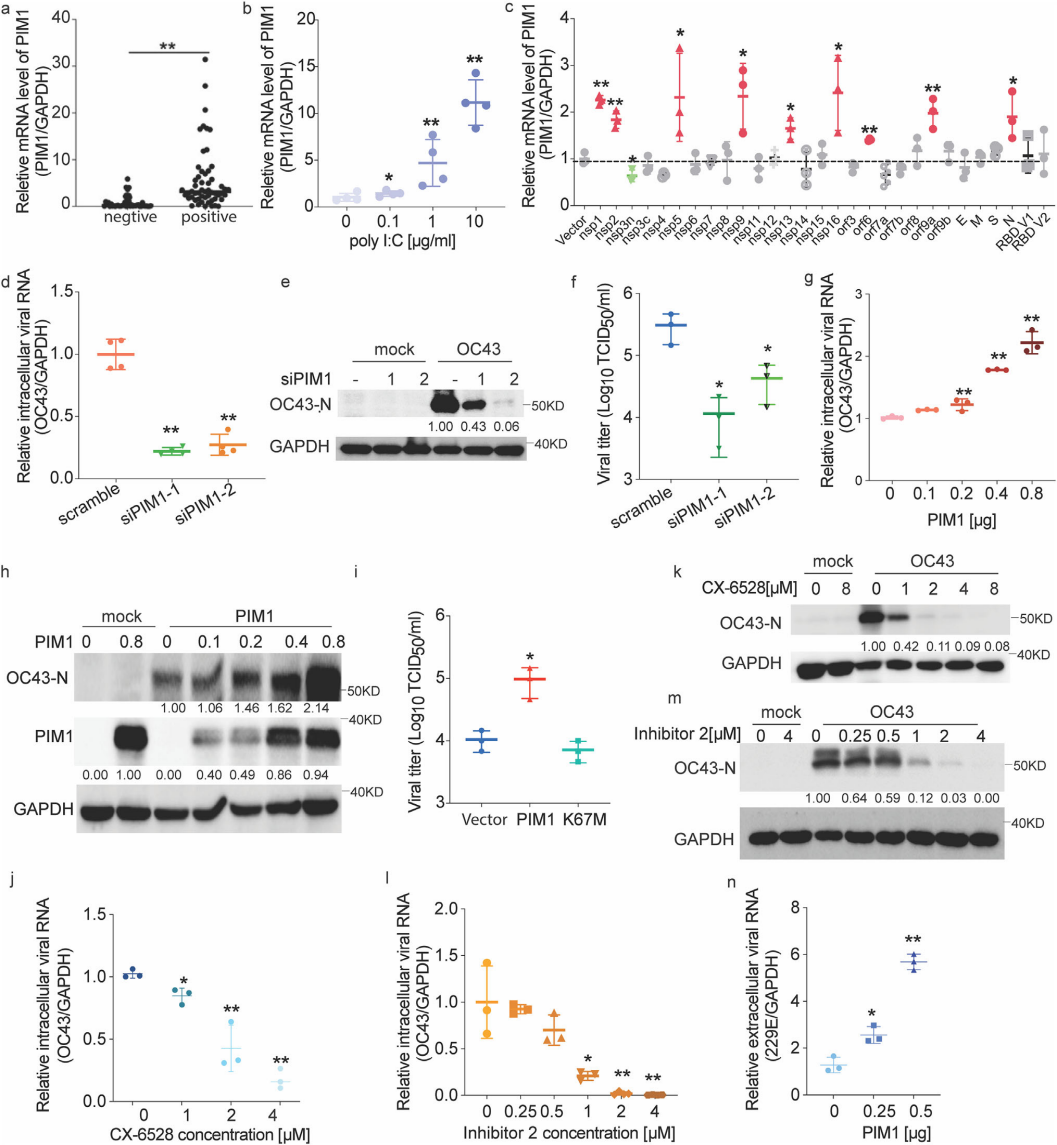
PIM1 was induced by HCoV infection and promoted HCoV replication. a–c) PIM1 mRNA levels in posterior pharyngeal wall cells from SARS‐CoV‐2 positive (n = 55) and negative (n = 46) subjects; RD cells transfected with poly I:C (n=4); HEK293T cells transfected with SARS‐CoV‐2 non‐structural, structural, and accessory proteins for 48 h (n = 3). d–f) OC43 viral genome RNA (n = 4), protein, and viral titer (n = 3) levels in PIM1‐knockdown HEK293T cells post infection. HEK293T cells were transfected with PIM1‐specific siRNAs for 24 h, then challenged with OC43 at an MOI of 1 for 24 h. g,h) Viral genome RNA g) (n = 3) and protein levels h) in HEK293T cells transfected with indicated amounts of PIM1 plasmids for 24 h, followed by OC43 infection at an MOI of 1 for 48 h. i) Viral titration in HEK293T cells were transfected with PIM1/PIM1 K67M plasmids for 24 h, followed by OC43 infection at an MOI of 1 for 24 h (n = 3). j–m) Viral genome RNA (n=3) and protein levels in virus‐challenged RD cells pretreated with PIM1 inhibitors. RD cells were pre‐treated with CX‐6528 or PIM1 inhibitor 2 for 2 h, followed by OC43 challenge at an MOI of 0.05 for 48 h. n) The intracellular viral genome RNA level of 229E after PIM1 modulation, followed by 48 h.p.i (n = 3). Statistical analyses were conducted with Student's t‐test. ^*^: *p* < 0.05, ^**^: *p* < 0.01. Data are shown as mean ±SD. All mRNA or viral genome RNA levels were determined by RT‐qPCR. Proteins were measured by western blot. Viral titration was determined by TCID50. The densities of all proteins of interest were quantified using ImageJ and normalized to the respective control indicated beneath the bands.

Upon infection with HCoV‐OC43, we found a significant boost of PIM1 expression exceeding tenfold in both A549 (Figure S1a, Supporting Information) and RD cells (Figure S1b, Supporting Information). Elevated PIM1 expression in COVID‐19 patients was observed as well (**Figure** **1**a). Replication‐deficient virus, treated with UV light, marginally increased PIM1 expression (Figure S1c, Supporting Information), suggesting viral components are crucial for the stimulation. Mimicking viral replication intermediates with poly: IC resulted in a dose‐dependent increase in PIM1 expression (Figure 1b). SARS‐CoV‐2 proteins, including non‐structural (nsp1, 2, 5, 9, 13, and 16), accessory (orf6, orf9a), and structural proteins (N)^[^ ^23^, ^24^ ^]^ significantly elevated PIM1 expression (Figure 1c). The expression levels of PIM2 and PIM3 also increased, but not as markedly as PIM1 during HCoV‐OC43 infection (Figure S1d,e, Supporting Information). Taken together, infection by β‐coronaviruses such as SARS‐CoV‐2 and OC43, along with their replication intermediates and specific viral proteins, upregulates PIM1 expression.

### 2.2 PIM1 Promotes Virus Replication in a Kinase‐Dependent Manner

To dissect the role of PIM1 in virus infection, we knocked down PIM1 in RD cells, followed by OC43 infection (Figure S1f, Supporting Information). PIM1 knockdown significantly decreased the OC43 viral RNA replication, viral protein level, and virus yield (Figure 1d–f). Supplementary PIM1 expression in PIM1‐knockdown cells restored virus replication (Figure S1g, Supporting Information). Ectopic expression of PIM1 increased OC43 replication in a dose‐dependent manner (Figure 1g,h) and potentiated virus yield (Figure 1i), but the PIM1 kinase‐dead mutation (K67M) failed to do so (Figure 1i). Furthermore, three pan‐PIM inhibitors (CX‐6258, SGI‐1776, and AZD‐1208) potently inhibited virus replication in a dose‐dependent manner (Figure 1j,k; Figure S1h–k, Supporting Information), indicating PIM1 kinase activity is crucial for virus replication. Furthermore, PIM1‐specific inhibitor (PIM1 inhibitor 2) showed a stronger effect on inhibiting virus replication (Figure 1l,m), evident by the lower concentration of inhibitor 2 required to block OC43 replication. Additionally, ectopic expression of PIM1 increased viral RNA levels of another β‐Coronavirus (HCoV‐229E) (Figure 1n). These results demonstrated that PIM1 was required for HCoV (OC43 and 229E) replication and could be a potential antiviral target.

### 2.3 PIM1 Downregulates IFNAR1 to Attenuate Cellular Innate Immunity

**Figure 2:**
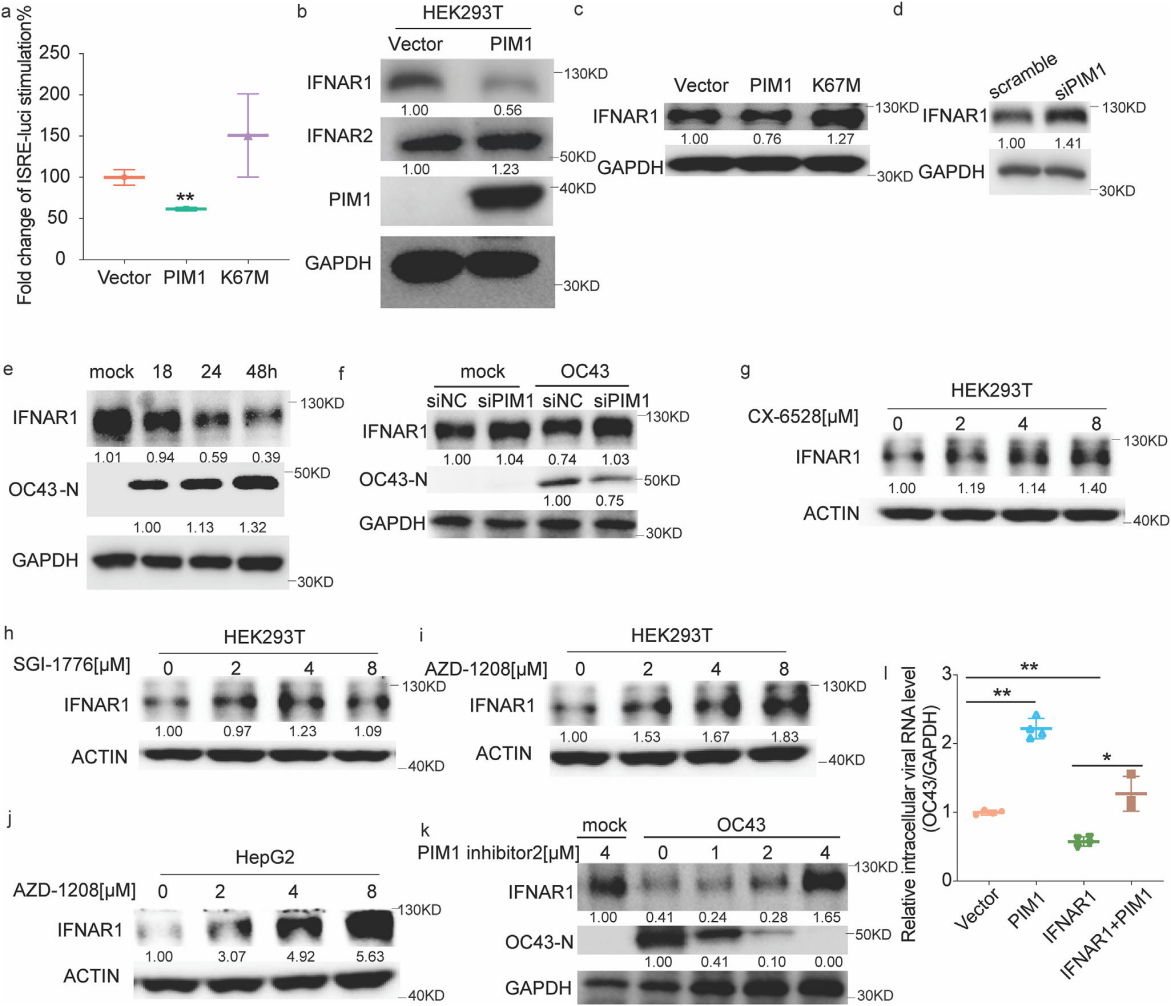
PIM1 decreased IFNAR1 protein levels to promote OC43 replication. a) Luciferase assay of ISRE element activity after PIM1/K67M overexpression (n = 3). b) Immunoblots of lysates from HEK293T cells transfected with vector/PIM1 plasmids for 48 h. c) Western blot for IFNAR1 in HEK293T cells transfected with vector/WT‐PIM1/PIM1‐K67M plasmids for 48 h. d) IFNAR1 expression level in PIM1 knockdown cells. e) Immunoblots analysis of RD cells infected with OC43 at an MOI of 10 for the indicated time. f) Expression of IFNAR1 in RD cells transfected with siPIM1 RNA, followed by OC43 infection. g–j) Western blot for IFNAR1 expression in HEK293T or HepG2 cells treated with PIM1 inhibitors (CX‐6528, SGI‐1776, and AZD‐1208) at the indicated concentration for 48 h. k) The IFNAR1 expression in RD cells pre‐treated with PIM1 inhibitor 2 for 2 h, followed by OC43 infection for 48h. l)The intracellular OC43 genomic RNA in HEK293T cells transfected with vector/PIM1/PIM1 with IFNAR1 followed by the 24 h challenging of OC43 at an MOI of 0.1. Statistical analyses were conducted with Student's t‐test. ^*^: *p* < 0.05, ^**^: *p* < 0.01. Data are depicted as mean ±SD. The densities of all proteins of interest were quantified using ImageJ and normalized to the respective control indicated beneath the bands.

Given PIM1's role in promoting viral replication and involvement in innate immunity,^[^ ^16^, ^17^, ^25^, ^26^ ^]^ we hypothesized that PIM1 enhanced viral replication by attenuating cellular innate immunity. Therefore, we examined the effects of PIM1 on the activities of interferon‐stimulated response element (ISRE) and interferon promoters. We co‐overexpressed PIM1 or PIM1‐K67M with ISRE‐luci, IFNα‐luci, or IFNβ‐luci reporters in HEK293T cells, and examined the luciferase activity 48 h post‐transfection. Only PIM1 decreased the promoter activity of ISRE‐luci/IFNα‐luci/IFNβ‐luci (**Figure** **2**a; Figure S2a,b, Supporting Information). Consistently, PIM1 kinase inhibitor dramatically increased the IFNβ‐luci stimulation (Figure S2c, Supporting Information). Ectopic expression of PIM1 decreased the phosphorylation level of STAT1(p‐STAT1) (Figure S2d, Supporting Information). However, PIM inhibitors (CX‐6258 and AZD‐1208) displayed a dose‐dependent increase of phosphorylated STAT1/STAT2 regardless poly I:C or IFNα stimulation (Figure S2e,f, Supporting Information). All these data indicated that PIM1 attenuates cellular innate immunity via downregulating the interferon signaling cascade.

We further examined the status of upstream interferon receptors IFNAR1 and IFNAR2 with ectopic expression of PIM1 or PIM1‐K67M in HKET93T cells. PIM1 specifically reduced IFNAR1 expression but not IFNAR2 at the protein level (Figure 2b), and the kinase‐dead PIM1 mutant increased IFNAR1 protein level (Figure 2c). Knocking down PIM1 had the opposite effect on IFNAR1 expression in HEK293T (Figure 2d). Interestingly, OC43 infection also downregulated IFNAR1 level in the host cells (Figure 2e). Most importantly, PIM1 knockdown prohibited virus infection‐induced IFNAR1 downregulation (Figure 2f). PIM1 kinase inhibitors increased IFNAR1 expression in a dose‐dependent manner in two different cell lines (Figure 2g–j). Significantly, PIM1‐specific inhibitor (PIM1 inhibitor 2) rescued virus‐induced IFNAR1 depletion (Figure 2k). After restoring IFNAR1 expression in the ectopic PIM1‐expressing cells, the PIM1‐promoted virus replication was effectively impeded (Figure 2l). Taken together, PIM1 facilitated virus replication by attenuation of cellular innate immunity through downregulation of IFNAR1.

### 2.4 PIM1 Downregulates IFNAR1 Through K63‐Linked Ubiquitination

**Figure 3:**
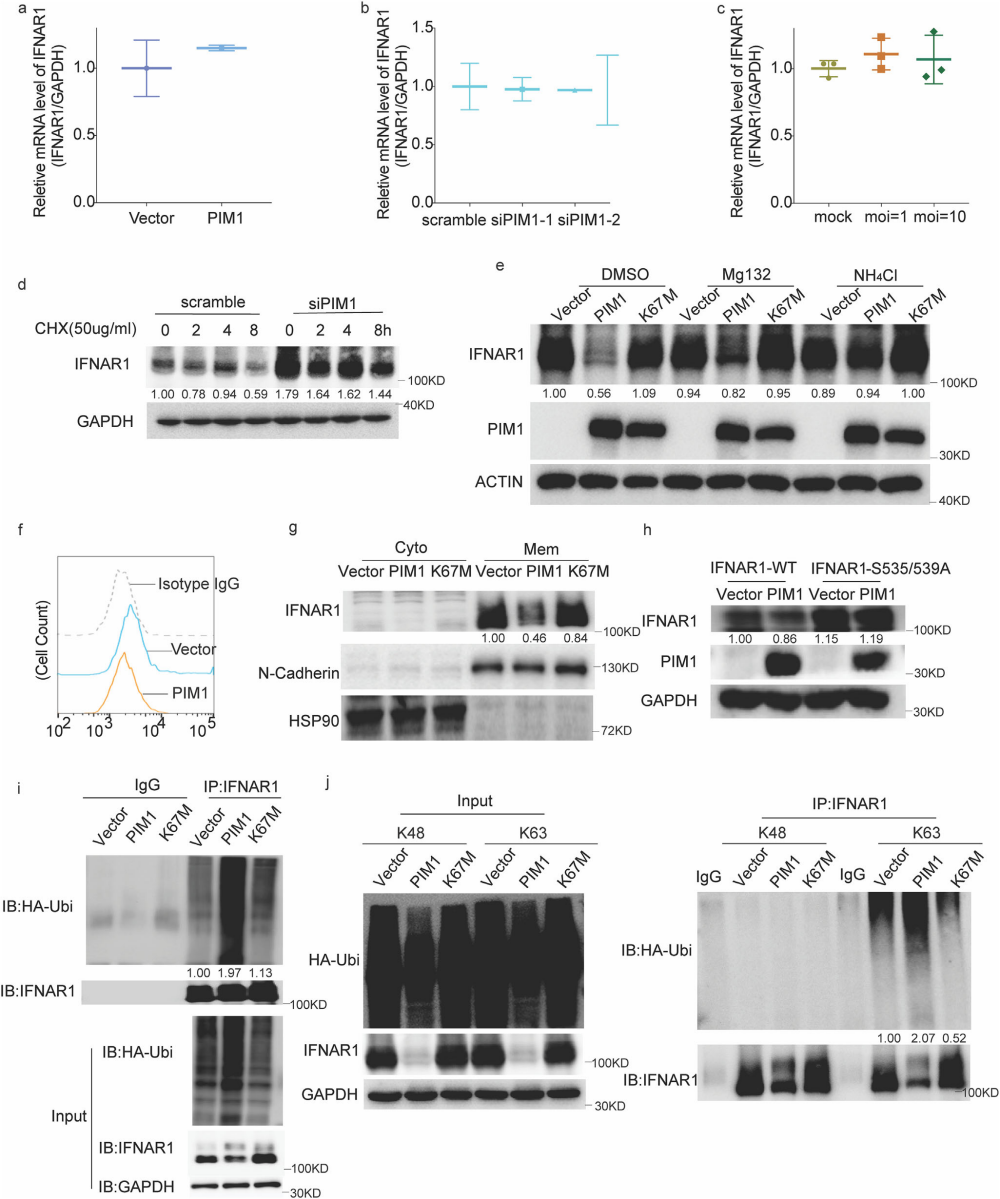
PIM1 promoted IFNAR1 degradation via ubiquitination. a) IFNAR1 mRNA level in HEK293T cells transfected with vector/PIM1 plasmids for 48 h. b) The mRNA level of IFNAR1 in RD cells transfected with scramble/PIM1‐specific siRNA for 48 h. c) The mRNA level of IFNAR1 in RD cells after challenging with/without OC43 at an MOI of 1 or 10. d) IFNAR1‐overexpressed HEK293T cells were transfected with scramble/PIM1‐specific siRNA, followed by 50 µm of CHX treatment for the indicated periods. IFNAR1 protein level were determined by western blot. e) IFNAR1 expression level after PIM1/K67M overexpression followed by MG132 or NH_4_Cl treatment. f) Flow Cytometry of cell surface IFNAR1 after PIM1 overexpression, stained with IFNAR1‐PE (n = 3). g) IFNAR1 expression in cell membrane or cytosol fractions after PIM1/K67M overexpression. h) Immunoblots of IFNAR1 in HEK293T cells transfected with WT‐IFNAR1 or mutated IFNAR1 (S535A, S539A) with/without PIM1. i) Western blot analysis for ubiquitination and IFNAR1 in HEK293T cells co‐transfected with HA‐Ubiquitin, IFNAR1 and vector/PIM1/PIM1 K67M constructs. j) Immunoprecipitation of IFNAR1 and HA‐tagged ubiquitination from lysates of HEK293T cells co‐transfected with HA‐K48 ubi/HA‐K63 ubi, IFNAR1 and vector/PIM1 plasmids. Statistical analyses were conducted with Student's t‐test. ^*^: *p* < 0.05, ^**^: *p* < 0.01. Data are depicted as mean ±SD. The densities of all proteins of interest were quantified using ImageJ and normalized to the respective control indicated beneath the bands.

To investigate how PIM1 downregulates IFNAR1 expression, we examined the effects of PIM1 on IFNAR1 transcription. Ectopic expression or knockdown of PIM1 did not affect the mRNA level of IFNAR1 (**Figure** **3**a,b). HCoV‐OC43 infection also failed to alter IFNAR1 transcription (Figure 3c). We subsequently examined the IFNAR1 protein turnover by using translation inhibitor cycloheximide (CHX) post‐siRNA‐depletion of PIM1, assuming that PIM1 may promote IFNAR1 degradation. Degradation of IFNAR1 was significantly slowed down in the PIM1 silencing group compared to the control group, as expected (Figure 3d). To further explore if PIM1 induced IFNAR1 downregulation via proteasome or lysosome degradation, MG132 and NH_4_Cl were used in PIM1/K67M overexpressed HEK293T cells, respectively. PIM1 caused IFANR1 degradation was rescued by NH_4_Cl treatment (Figure 3e), indicating the involvement of lysosomal degradation.

IFNAR1 is primarily resident on the cell membrane and is known to be degraded by lysosomes. We hence employed a flow cytometry assay, which showed that PIM1 overexpression dramatically down‐regulated surface IFNAR1 expression (Figure 3f). So did the cell membrane fraction assay (Figure 3g). It was reported that the lysosomal degradation of surface IFNAR1 undergoes S535/S539 phosphorylation, which promotes its ubiquitination and subsequent internalization upon exposure of the internalization motif.^[^ ^27^ ^]^ As a threonine/serine kinase, PIM1 was speculated to modulate IFNAR1 phosphorylation. However, ectopic expression of PIM1 reduced the IFNAR1 phosphorylation level (Figure S3a, Supporting Information), whereas PIM1 knockdown or PIM1 inhibitor increased the IFNAR1 phosphorylation level (Figure S3b–d, Supporting Information), excluding the possibility of IFNAR1 as a PIM1 substrate. Moreover, the Co‐immunoprecipitation assay (Co‐IP) revealed that PIM1 failed to interact with IFNAR1 (Figure S3e, Supporting Information), again indicating that PIM1 has no direct influence on the phosphorylation of IFNAR1.

Intriguingly, PIM1 only downregulated the wild‐type IFNAR1 but not the phosphorylation‐deficient (S535/S539A) IFNAR1 mutant (Figure 3h), indicating that the downregulation of IFNAR1 by PIM1 is still phosphorylation‐dependent, even though the process is not related to PIM1 kinase activity. We subsequently examined the status of IFNAR1 ubiquitination, a key modification for IFNAR1 endocytosis and degradation.^[^ ^4^ ^]^ Ectopic PIM1 expression potently increased the IFNAR1 ubiquitination level (Figure 3i). Further data revealed that PIM1 induced K63‐linked but not K48‐linked or other Lysine‐linked ubiquitination of IFNAR1 (Figure 3j; Figure S3f, Supporting Information), indicating that PIM1 promotes IFNAR1 degradation via K63‐linked ubiquitination. This result is consistent with the report that K63‐linked ubiquitination is important for surface IFNAR1 exposure of its endocytosis motif.^[^ ^5^, ^7^, ^28^ ^]^

### 2.5 PIM1 Phosphorylates β‐TrCP1 to Ubiquitinate IFNAR1

**Figure 4:**
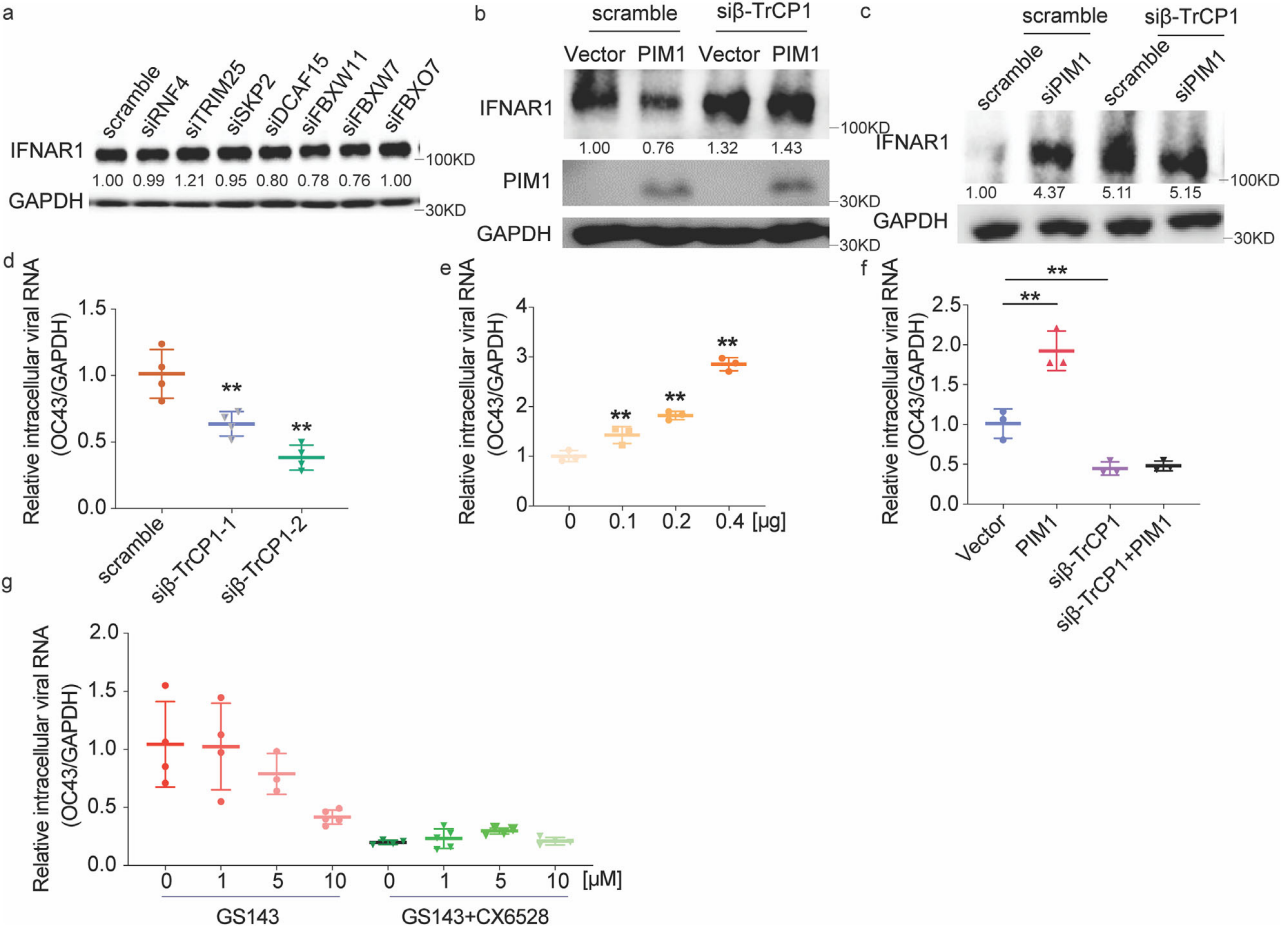
PIM1 promoted IFNAR1 degradation via β‐TrCP1. a) IFNAR1 protein level in IFNAR1‐expressed HEK293T cells transfected with diverse siRNAs. b) IFNAR1 protein level in IFANR1‐expressed HEK293T cells transfected with scramble/β‐TrCP1 specific siRNA with/without PIM1 plasmids. c) IFNAR1 protein level in IFNAR1‐expressed HEK293T cells with β‐TrCP1 and/or PIM1 siRNA knockdown. d) Viral genomic RNA of OC43 in RD cells transfected with scramble/β‐TrCP1 specific siRNA followed by infection at an MOI of 0.1 for 24 h (n = 4). e) Viral genomic RNA level of OC43 in HEK293T cells transfected with increasing amounts of β‐TrCP1 followed by the challenging of HCoV‐OC43 at an MOI of 0.1 for 24 h (n = 3). f) Viral genomic RNA level in HEK293T cells after β‐TrCP1 siRNA knockdown with or without ectopic expression of PIM1, followed by OC43 infection at an MOI of 0.1 for 24 h (n = 3). g) Viral genomic RNA level in RD cells treated with PIM1 inhibitor (CX6528=8 µm) with or without β‐TrCP1 inhibitor (GS143) at indicated concentration for 2 h, followed by OC43 infection at MOI of 0.01 for 72 h (n = 4). Statistical analyses were conducted with Student's t‐test. ^*^: *p* <0.05, ^**^: *p* < 0.01. Data are shown as mean ±SD. The densities of all proteins of interest were quantified using ImageJ and normalized to the respective control indicated beneath the bands.

There are over 1000 E3 ubiquitin ligases that extensively regulate cell cycle, cell trafficking, DNA damage, cell signaling, and innate immunity in mammalian cells.^[^ ^29^ ^]^ To identify the E3 ligase that promoted the PIM1‐induced IFNAR1 degradation, seven candidates were obtained via bioinformatics prediction using UbiBrowser (v.2),^[^ ^30^ ^]^ including β‐TrCP2 (FBXW11), RNF4, TRIM25, SKP2, DCAF15, FBXW7, and FBXO7. We knocked them down one by one and, disappointedly, found out that only TRIM25 depletion slightly increased the IFNAR1 level (**Figure** **4**a; Figure S4a, Supporting Information). However, literature showed that β‐TrCP1, an E3 ligase that is known to ubiquitinate phosphorylated substrate, was involved in IFNAR1 ubiquitination.^[^ ^5^ ^]^ By knockdown of β‐TrCP1 in ectopic PIM1‐expressing cells, it not only abolished PIM1‐induced IFNAR1 reduction but also increased IFNAR1 levels (Figure 4b). Consistently, knockdown of PIM1 or β‐TrCP1 or both elevated IFNAR1 expression (Figure 4c). Furthermore, knockdown of β‐TrCP1 significantly suppressed HCoV‐OC43 replication (Figure 4d; Figure S4b, Supporting Information) while ectopic expression of β‐TrCP1 dramatically promoted HCoV‐OC43 virus replication (Figure 4e). Depletion of β‐TrCP1 eliminated PIM1‐promoted HCoV‐OC43 replication (Figure 4f). Cotreatment with β‐TrCP1 inhibitor GS143 and PIM1 inhibitor CX‐6528 had no additional stack effect against virus replication compared to individual inhibitors (Figure 4g), suggesting that PIM1 and β‐TrCP1 work together to coordinate IFNAR1 degradation in the same pathway.

### 2.6 PIM1 Phosphorylates β‐TrCP1 to Form β‐TrCP1/IFNAR1 Complex

**Figure 5:**
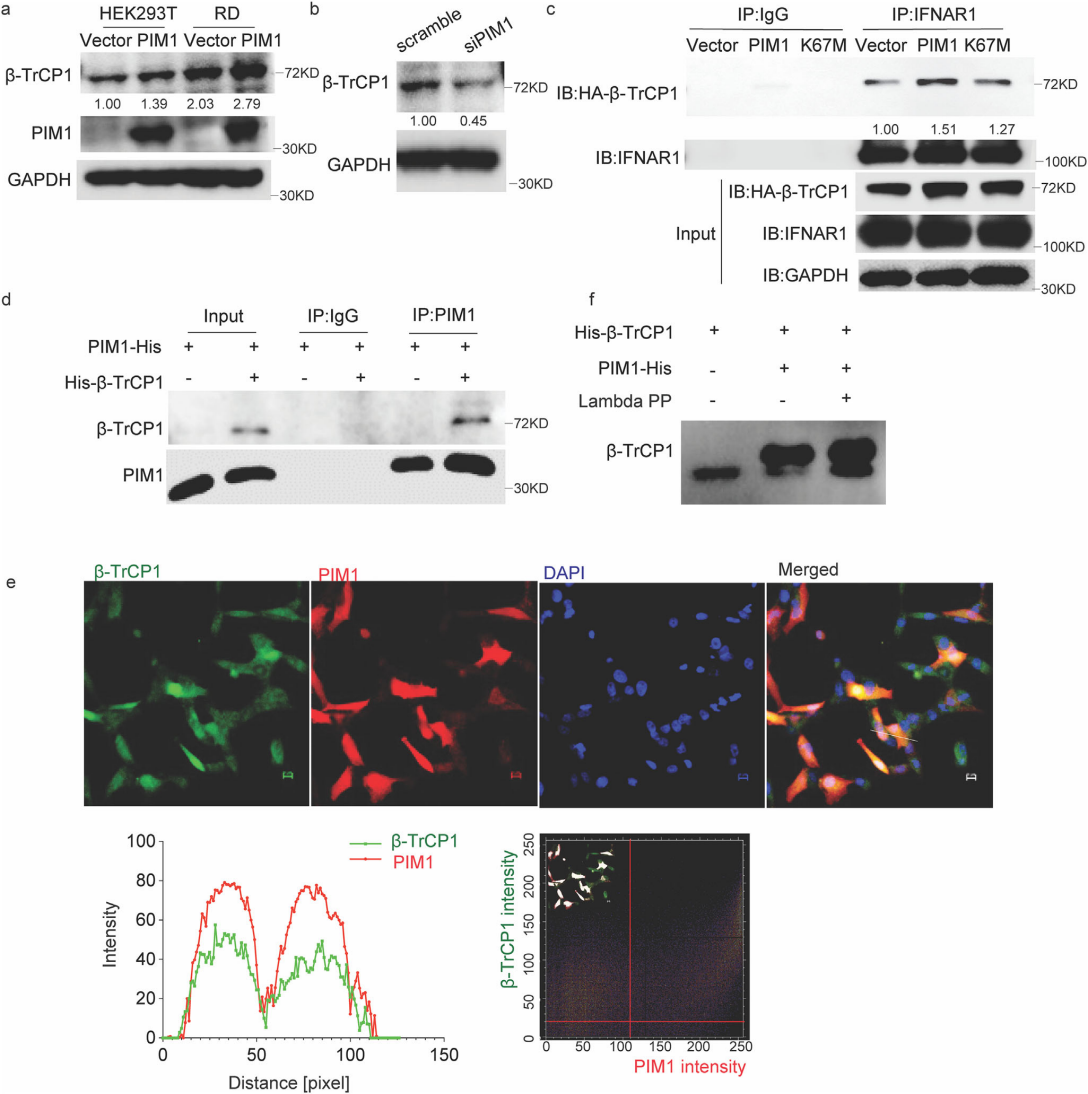
PIM1 promoted IFNAR1 and β‐TrCP1 interaction. a,b) Protein level of β‐TrCP1 in HEK293T cells transfected with vector/PIM1 construct or scramble/PIM1 specific siRNA. c) Immunoprecipitation of IFNAR1 and HA‐tagged β‐TrCP1 from lysates mixture of vector/PIM1/PIM1 mutant expressed HEK293T and IFNAR1 expressed HEK293T. d) IP blots for purified PIM1 post incubation with or without purified βTrCP1 in the IP buffer. e) Immunofluorescent assay of HEK293T cells co‐transfected with PIM1 and HA‐ β‐TrCP1 plasmids under 40× microscope. PIM1 was in Red and HA‐ β‐TrCP1 was in Green. Scale bar = 10 µm. Line intensity profiles (indicated as white line) and Scatter plots (boxed ROI ) for PIM1 and β‐TrCP1 were analyzed by Image J software. Pearson's r = 0.62. f) Purified β‐TrCP1 incubated with/without recombinant PIM1 in a kinase assay buffer at 37 °C for 30 min. Lambda protein phosphatase (lambda PP) was added to one tube of the PIM1 and β‐TrCP1 mixture for another 30 min. The products were visualized post Phos‐tag PAGE separation, which separates the phosphorylated β‐TrCP1 as up‐shifted migration bands from the non‐phosphorylated ones. The densities of all proteins of interest were quantified using ImageJ and normalized to the respective control indicated beneath the bands.

Neither PIM1 knockdown nor PIM1 inhibitors affected the mRNA level of β‐TrCP1 (Figure S4c,d, Supporting Information). HCoV‐OC43 infection also did not affect β‐TrCP1 transcription (Figure S4e, Supporting Information). In contrast, PIM1 increased the β‐TrCP1 protein level in HEK293T cells and RD cells (**Figure** **5**a), while PIM1 knockdown significantly decreased it (Figure 5b). Similarly, PIM1 inhibitors decreased β‐TrCP1 protein level (Figure S4f,g, Supporting Information). All these results indicated that PIM1 stabilized β‐TrCP1 protein. Subsequently, we investigated whether PIM1 affects the interaction between β‐TrCP1 and its substrate IFNAR1. PIM1 but not the kinase‐dead PIM1 significantly enhanced the interaction of IFNAR1 with β‐TrCP1 (Figure 5c).

We confirmed interaction between β‐TrCP1 and PIM1 via bidirectional Co‐IP assay (Figure S4h,i, Supporting Information). To rule out the potential involvement of other intermediating molecules, we used purified recombinant PIM1 and β‐TrCP1 in subsequent experiments. The result of the in vitro experiment showed that PIM1 directly formed a complex with β‐TrCP1 (Figure 5d). Immunofluorescent assay revealed that PIM1 co‐localized with β‐TrCP1 in RD cells (Figure 5e). We therefore assumed that β‐TrCP1 is a substrate of PIM1. As expected, the kinase activity assay revealed that the recombinant PIM1 directly phosphorylated βTrCP1, of which phosphorylation could be removed by Lambda protein phosphatase (Figure 5f).

### 2.7 Phosphorylation of β‐TrCP at S82 Enhances β‐TrCP1/IFNAR1 Complex Formation

**Figure 6:**
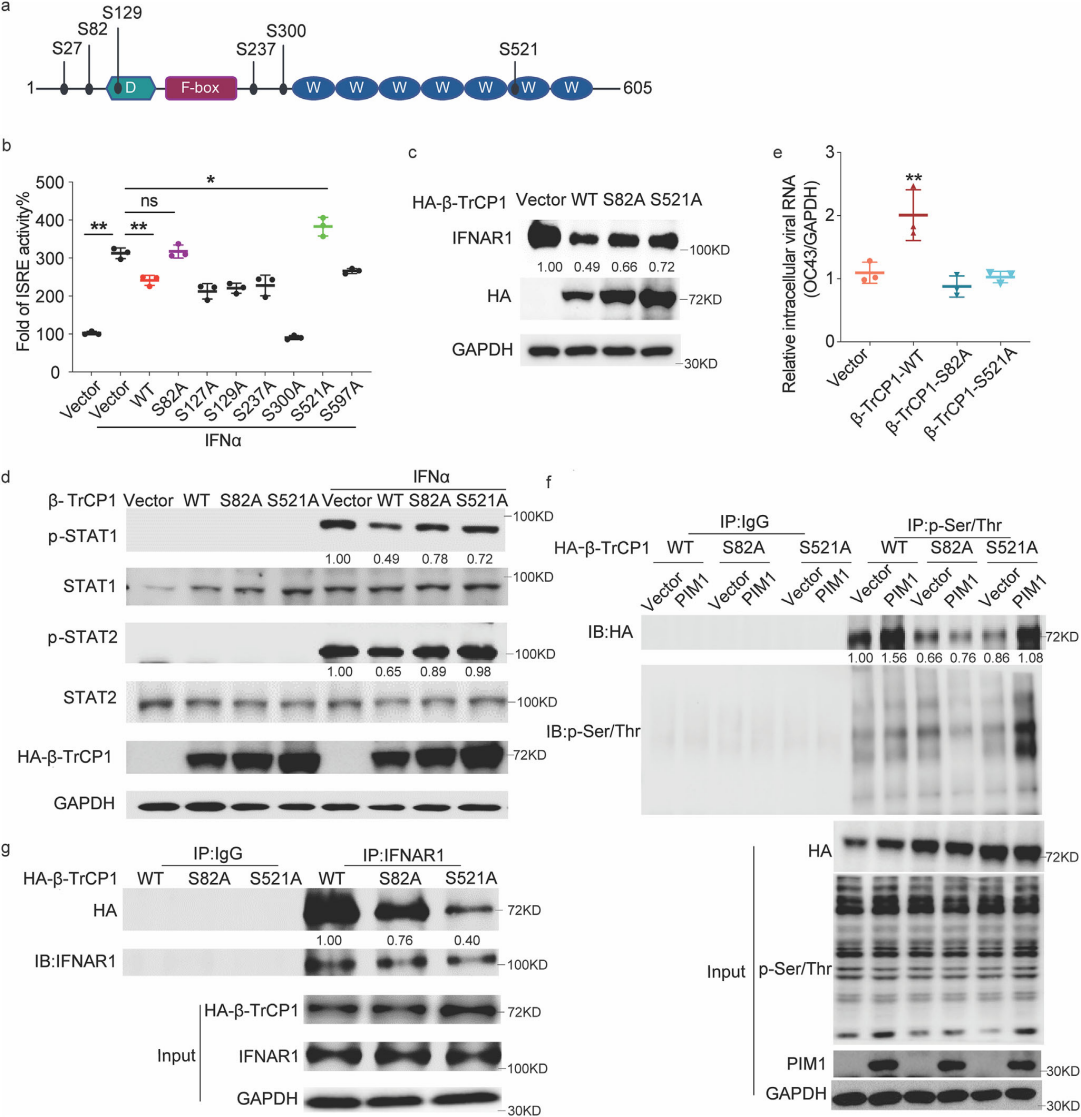
Phosphorylated β‐TrCP1 at S82 by PIM1 enhanced its interaction with IFNAR1. a) Predicted PIM1‐phosphorylation sites on β‐TrCP1. b) ISRE luciferase assays using HEK293T cells transfected with ISRE‐Luci and β‐TrCP1 / β‐TrCP1 mutants, followed by stimulation with 500U IFNα for 16 h (n = 3). c) Protein level of IFNAR1 in HEK293T cells ectopically expressed with WT‐ β‐TrCP1 / β‐TrCP1 S82A/ β‐TrCP1 S521A. d) Protein level of phosphorylated STAT1/2 (p‐STAT1/2) in HEK293T cells ectopically expressed with vector/WT‐ β‐TrCP1 / β‐TrCP1 S82A/ β‐TrCP1 S521A followed by the stimulation of IFNα for 16 h. e) Quantification of intracellular viral genomic RNA in HEK293T cells ectopically expressed with WT‐ β‐TrCP1 / β‐TrCP1 S82A/ β‐TrCP1 S521A followed by the infection with HCoV‐OC43 at an MOI of 1 for 36 h (n = 3). f) Western blot analysis of HEK293T cells transfected with WT/S82A/S521A β‐TrCP1 plasmids with/without PIM1. Blots of IP assay using p‐Ser/Thr antibody were shown in the bottom panel. g) Co‐IP assay of IFNAR1 from the cell lysate mixture of HEK293T cells transfected with IFNAR1/HA‐ β‐TrCP1 / β‐TrCP1 S82A/ β‐TrCP1 S521A separately.

Predicted by NetPhos 3.1 program,^[^ ^31^ ^]^ PIM1 may phosphorylate 7 serine residues (S82, 127, 129, 237, 300, 521, and 597) of βTrCP1 (**Figure** **6**a). We mutated each of the residues into alanine (i.e., S82A, S127A, S129A, S237A, S300A, S521A, or S597A) and tested their effects. As compared with WT‐β‐TrCP1, S82A, and S521A β‐TrCP1 mutants failed to decrease ISRE activity (Figure 6b), while they partially restored IFNAR1 expression (Figure 6c) and the STAT1/STAT2 phosphorylation (Figure 6d) upon IFNα stimulation. In addition, β‐TrCP1 significantly enhanced HCoV‐OC43 replication whereas both S82A and S521A mutants failed to do so (Figure 6e). Although both S82A and S521A mutants displayed a higher expression level, to our surprise, PIM1 failed to enhance the phosphorylated level of mutant β‐TrCP1(S82A) (Figure 6f), which was further confirmed by quantitative mass spectrum assay (Figure S5a–f, Supporting Information).

We further assessed whether the S82 phosphorylation of β‐TrCP1 affects its substrate binding. S521 of β‐TrCP1 is close to the ADP‐parylation modification motif of β‐TrCP1, which affects the substrate binding ability of β‐TrCP1, including IFNAR1.^[^ ^5^ ^]^ After replacing S82 and S521 with alanine, the interaction between IFNAR1 and β‐TrCP1 significantly decreased (Figure 6g). All these data demonstrated that PIM1 phosphorylates S82 of β‐TrCP1, leading to β‐TrCP1/IFNAR1 complex formation and IFNAR1 ubiquitination.

## 3 Discussion

Viruses have evolved several strategies to downregulate IFNAR1 and promote their replication. These include transcriptional inhibition via microRNAs by HCV,^[^ ^32^ ^]^ impairment of glycosylation to disrupt IFNAR1 maturation by flaviviruses^[^ ^10^ ^]^ induction of extracellular inhibitory ligands for IFNAR1 by viral proteases,^[^ ^7^ ^]^ and acceleration of IFNAR1 degradation by enhancing its recognition by E3 ubiquitin ligases, often via phosphorylation at residues S535/S539 on IFNAR1 itself.^[^ ^33^, ^34^, ^35^ ^]^ Here, we discovered the critical role of PIM1 in promoting human β‐coronavirus replication, including HCoV‐OC43 and HCoV‐229E, via a novel type of IFNAR1 regulation mechanism. PIM1 and OC43 infection do not affect the mRNA level of IFNAR1, which is consistent with that of clinical blood samples of COVID‐19 patients evaluated by another group.^[^ ^36^ ^]^ Instead, PIM1 decreases the cell surface expression of IFNAR1. Without directly manipulating the posttranslational modifications (PTMs) status of IFNAR1, SARS‐CoV‐2, and HCoV‐OC43 upregulated host kinase PIM1 (Figure 1). PIM1, in turn, phosphorylates the host E3 ubiquitin ligase β‐TrCP1, enhancing its affinity to IFNAR1 (Figures 5 and 6). Subsequently, PIM1 induces IFNAR1 K63‐linked ubiquitination and degradation. It is reported that this kind of ubiquitination of IFNAR1 causes endocytosis motif exposure and subsequent internalization and lysosomal degradation of IFNAR1.^[^ ^4^, ^27^ ^]^ Our data shows that PIM1‐induced IFNAR1 decrease could be rescued by lysosome inhibitor NH_4_Cl, which is consistent with previous reports. Our findings reveal a novel layer of regulation regarding IFNAR1 stability, highlighting a complex post‐translational modification (PTM) network that viruses manipulate to evade the host immune response.

Kinases participate in the regulation of almost every cellular physiological event, including the host response to viral infection. Significant perturbations of the kinase network‐especially those associated with immune responses ‐ were documented in coronavirus‐infected cells.^[^ ^37^ ^]^ However, the precise mechanisms through which kinases aid coronaviruses in circumventing innate immunity remain elusive. PIM1 kinase, previously implicated in facilitating transcription of several (+) ssRNA viruses,^[^ ^17^, ^25^, ^26^, ^38^ ^]^ has an unclear role in human β‐coronaviruses. In the presented study, we showed that PIM1 expression was activated in COVID‐19 patients, and its kinase activity was required to promote replication of human β‐coronaviruses, HCoV‐OC43 (Figure 1). Of significance, HCoV‐OC43 infection induces PIM1, 2, and 3 increases, with PIM1 changes more remarkably. And PIM1‐specific inhibitor (PIM1 inhibitor 2) has a stronger antiviral effect than pan‐PIM inhibitors (CX‐6528, SGI‐1776, and AZD‐1208). All these results indicated that PIM1 is the main PIM in this family to foster virus replication. Importantly, we demonstrated for the first time that PIM1 suppressed IFNAR1 but not IFNAR2 (Figure 2). Our discovery thus provides novel insights into bridging the modulation of IFNAR1‐mediated innate immune response with the coronavirus‐perturbed kinase network. Given its established role in promoting cell survival and anti‐apoptotic,^[^ ^14^, ^39^ ^]^ activation of PIM1 not only represses the interferon responses but may also prolong the survival of infected cells. The proposed PIM1‐mediated IFNAR1 degradation mechanism, therefore, could at least partially explain the inverse correlation of serum IFNAR1 level with COVID‐19 severity,^[^ ^13^ ^]^ and indicates a potential therapeutic value of PIM1 kinase that is supported by our data (Figure 2).

Protein phosphorylation is widely employed by the protein degradation mechanism as a “tag” for substrate recognition and crosstalk with other PTMs, particularly ubiquitination.^[^ ^40^, ^41^, ^42^ ^]^ Our research reveals that PIM1 does not directly phosphorylate IFNAR1 (Figure S3, Supporting Information), but instead targets Serine 82 residue of E3 ligase β‐TrCP1, enhancing its substrate affinity (Figure 6; Figure S5, Supporting Information). Such phosphorylation events on E3 ligases have been recently recognized to affect their functions, including enzymatic activation, cognate binding alteration,^[^ ^43^ ^]^ or changes in substrate preference.^[^ ^44^ ^]^ Our findings contribute to the understanding of how PTMs on E3 ligase regulate its function and influence substrate affinity. Interestingly, PIM1 does not regulate β‐TrCP1 transcriptionally (Figure S4, Supporting Information), suggesting that PIM1's effect is post‐translational. This conclusion is supported by the fact that no additional effects were observed upon combinatory treatments of PIM1 and β‐TrCP1 inhibitors (Figure 4g). Consequently, we identify β‐TrCP1 as a novel substrate of PIM1, establishing a regulatory axis at a posttranslational level between PIM1, β‐TrCP1, and IFNAR1. Notably, we also found another serine residue S521, served a crucial role in β‐TrCP1/IFNAR1 complex formation without being phosphorylated by PIM1 (Figure 6). Intriguingly, S521 is next to the PARylation motif (AA513‐520) of β‐TrCP1 that regulates its interaction with IFNAR1.^[^ ^5^ ^]^ Potential crosstalk between phosphorylation and PARylation might occur on S521 of β‐TrCP1 to regulate its substrate affinity, explaining why S521 is involved in its interaction with IFNAR1 without being phosphorylated by PIM1.

In conclusion, we elucidated a novel mechanism employed by human β‐coronavirus to compromise host innate immunity, of which viral induction of PIM1 kinase manipulates the PTM status of β‐TrCP1 to increase its affinity to IFNAR1. Our discovery bridges PIM1‐mediated PTM crosstalk that occurred on E3 ligase and interferon response, underscoring the delicate balance of viral replication and host signaling network. PIM1 kinase inhibitors both specific and pan‐PIM1 inhibitors potently inhibit viral replication (Figure 1), indicating PIM1 is a promising therapeutic target for further anti‐virus drug development.

## 4 Experimental Section

### Cells and Viruses

HEK293T cells (ATCC CRL‐3216), A549 cells (ATCC CCL‐185), human muscle rhabdomyosarcoma (RD) cells (ATCC CCL‐136), and Vero cells (ATCC CCL‐81) were cultured in Dulbecco's modified Eagle's medium (DMEM) containing 10% fetal bovine serum (FBS) with 100 U mL^−1^ penicillin and 100 µg mL^−1^ streptomycin. HCoV‐OC43 (VR‐1558) was obtained from ATCC. The amplification of HCoV‐OC43 was incubated in Vero cells with 2% FBS, 100 U mL^−1^ penicillin, and 100 µg mL^−1^ streptomycin in DMEM medium. HCoV‐OC43 was incubated with HEK293T, RD, or A549 cells at the indicated MOI and time point with 2% FBS, 100 U mL^−1^ penicillin, and 100 µg mL^−1^ streptomycin DMEM medium. HCoV‐229E (VR740) was propagated in MRC‐5 cells (ATCC CCL‐171). And the infection procedure was the same as that of HCoV‐OC43. Posterior pharyngeal wall cells were from clinical samples of COVID‐19 patients and negative control donors. All experiment protocols about pharyngeal wall cells were approved by the Weihai Municipal Hospital Ethics Committee (Approval Number: 20240064), and prior consent from human participants was obtained before sample collection.

### Plasmid Construction

Human PIM1 (NM_001243186) cDNA and IFNAR1 (NM_000629.3) were cloned into pcDNA4/HisMax B (Invitrogen, USA) vector between BamHI and XbarI sites. PIM1 mutant K67M (kinase dead PIM1) and IFNAR1 S535A and S539A plasmids were generated using the site mutation method. The primer information is as described before.^[^ ^17^ ^]^ K48‐Ub, K63‐Ub, and ISRE‐luci plasmids were gifts from Prof. Yuan.^[^ ^45^ ^]^ HA‐tagged‐β‐TrCP1 along with its site‐mutated plasmids, and K27R, K29R, K33R, K48R, K63R ubiquitination plasmids were custom‐made by Wuhan Miaoling Biotech Science Co Ltd. pGL3‐IFNβ (Cat:102597) plasmids were purchased from Addgene (USA).

### Antibodies and Reagents

The following antibodies were used in this project: rabbit anti‐IFNAR1 (Abcam, ab45172), rabbit anti‐IFNAR2 (sc‐137209), rabbit anti‐β‐TrCP (A18232), mouse anti‐HA‐tag (sc‐7392), rabbit anti‐STAT1 (Abcam, ab47425), rabbit anti‐p‐STAT1 (Abcam, ab30645), rabbit anti‐STAT2 (Santa Cruz, sc‐514193), rabbit anti‐p‐STAT2 (Abcam, ab53132), rabbit anti‐pSer/Thr (Thermo, PP2551), mouse anti‐ubiquitin (Santa Cruz, sc‐8017), rabbit anti‐PIM1 (CST, 3247), mouse‐anti‐GAPDH (Santa Cruz, sc‐47724), Human IFN‐alpha / beta R1 PE‐conjugated Antibody(R&D systems, FAB245P), rabbit anti‐N‐Cadherin (Abcam, ab76011), rabbit anti‐Hsp90 antibody(CST, 4877S), and mouse anti‐actin. Recombinant human IFN‐α and IFN‐β were obtained from PeproTech (Rocky Hill, USA).

PIM1 inhibitors were purchased from MCE (CX6528: HY‐18095; AZD‐1208: HY‐15604; SGI‐1776: HY‐13287; PIM1 inhibitor 2:^[^ ^46^ ^]^ HY‐147785). Transfection of plasmids was achieved using HD Fugene, which was purchased from Promega (USA). Transfection of siRNA was accomplished by using Hiperfect, which was purchased from QIAGEN (Germany). siRNAs were purchased from Genepharma (Shanghai, China). siRNA sequence information was as follows (**Table** **1**):

**Table 1:** siRNA sequence information.

Primer Name	Sequence
si‐PIM1‐1‐sense	5′‐AACCUUCGAAGAAAUCCAGAACCAU‐3′,
si‐PIM1‐1‐antisense	5′‐AUGGUUCUGGAUUUCUUCGAAGGUU‐3′;
siPIM1‐2‐sense	5′‐ACAUUUACAACUCAUUCCA‐3′,
siPIM1‐2‐antisense	5′‐UGGAAUGAGUUGUAAAUGU‐3′;
siPIAS2‐sense	5′‐CCUGUCCAUCCUGAUGUGCAGUUAA‐3′,
siPIAS2‐antisense	5′‐UUAACUGCACAUCAGGAUGGACAGG‐3′;
siβ‐TrCP1‐1‐sense	5′‐CGGAAACUCUCAGCAAGCUAUGAAA‐3′,
siβ‐TrCP1‐1‐antisense	5′‐UUUCAUAGCUUGCUGAGAGUUUCCG‐3′;
siβ‐TrCP1‐2‐sense	5′‐GAGCUGCUGUCAAUGUUGUAGACUU‐3′,
siβ‐TrCP1‐2‐antisense	5′‐AAGUCUACAACAUUGACAGCAGCUC‐3′;
sitrim25‐sense	5′‐GGGAUGAGUUCGAGUUUCUGGAGAA‐3′,
sitrim25‐antisense	5′‐UUCUCCAGAAACUCGAACUCAUCCC‐3′;
siRNF4‐sense	5′‐UAUGGGUUCUGCUUCCAAGGAGAUC‐3′,
siRNF4‐antisense	5′‐GAUCUCCUUGGAAGCAGAACCCAUA‐3′;
siSKP2‐sense	5′‐GCGACUUAACCUUUCUGGGUGUUCU‐3′,
siSKP2‐antisense	5′‐AGAACACCCAGAAAGGUUAAGUCGC‐3′;
siFBXO7‐sense	5′‐CCAAUCAGACUAGCAUGCAGGAUGA‐3′,
siFBXO7‐antisense	5′‐UCAUCCUGCAUGCUAGUCUGAUUGG‐3′;
siDCAF15‐sense	5′‐GAUGAGUUGGAGGACGACAAGAUCU‐3′,
siDCAF15‐antisense	5′‐AGAUCUUGUCGUCCUCCAACUCAUC‐3′;
siFBXW11‐sense	5′‐CGAGUGAUCUCAGAAGGAAUGCUUU‐3′,
siFBXW11‐antisense	5′‐AAAGCAUUCCUUCUGAGAUCACUCG‐3′.

### RNA Extraction and Real‐Time Quantitative PCR (RT‐qPCR)

Total cell RNA was isolated using Trizol reagent (Ambion, Life, Technologies) according to the manual. Subsequently, 1 µg of RNA was subjected to reverse transcription using the reverse transcription kit purchased from TAKARA. cDNA was further used for Quantitative Real‐time PCR (qRT‐PCR) with SYBR Green Mix (TAKARA). Each experiment was repeated triple, and every target gene expression was normalized by GAPDH expression. Data analysis of the relative quantification of gene expression was conducted by using the 2‐ΔΔCT method.

The primers used in RT‐qPCR are listed in **Table** **2**.

**Table 2:** The RT‐qPCR primers.

Primer Name	Sequence (5'‐3')
GAPDH F	GTCTCCTCTGACTTCAACAGCG
GAPDH R	ACCACCCTGTTGCTGTAGCCAA
PIM1 F	TTCATCACGGAAAGGGGAGC
PIM1 R	GGCCCCTGATCTCTTCG
IFNAR1 F	ATTTACACCATTTCGCAAAGCTC
IFNAR1 R	TCCAAAGCCCACATAACACTATC
OC43 F	TTGTGAGCGATTTGCGTGCG
OC43 R	ACACGTCCCTGGCTGAAAGC
TrCP‐1 F	ACCAACATGGGCACATAAACTC
TrCP‐1 R PIM2 F PIM2 R PIM‐3 F PIM3 R RNF4 F RNF4 R TRIM25 F TRIM25 R SKP2 F SKP2 R DCAF15 F DCAF15 R FBXW11 F FBXW11 R FBXO7 F FBXO7 R 229E F 229E R	TGGCATCCAGGTATGACAGAAT ACTGACTTTGATGGGACAAGGG AATGTCCCCACACACCATGT AAGGACGAAAATCTGCTTGTGG CGAAGTCGGTGTAGACCGTG GGTGGAGCAATAAATTCTAGACAAGC CCACCACAGGCTCTAAAGATTCACAAGTGAGG AATCGGCTGCGGGAATTTTTC TCTCACATCATCCAGTGCTCT TTCGGATCCCATTGTCAATACTC CAAAGTCTGCAGGGCAAATTC GAGGTCTGCCCAGAAACCAA CTCAGTCAGGTCGCCTACA‐C CGGGACTTTATCACTGCTTTA ATCACTCGCTGCCATTCTTTA AGTCCCTGCTGTGCACCTG CGCTGGAATGTCATCTTGAAGA CCCCCATGAGCAACATGAC GCCCATTTGACTGTAGCCATC

### Luciferase Assay

HEK 293T cells were plated in a 24‐well plate one day before transfection. The reporter vector of ISRE/IFNα/β together with the indicated WT or mutated expressing vector of PIM1/β‐TrCP1 were transfected into HEK293T cells for 24 h. Then the cells were stimulated with/without 1 µg of poly:IC or 500U of IFNα. 24 h post‐stimulation, cells were harvested using a passive lysis buffer (Promega, USA). Luciferase activities were measured with a luciferase assay kit (Promega, Madison, USA) according to the manufacturer's instructions.

### Western Blot

Cells were washed with PBS and harvested with lysis buffer (50 mm Tris Tris‐HCl, [pH 7.4], 150 mm NaCl, 1%Triton‐x100, 0.1% SDS, 1× protease inhibitor cocktail (MCE)). Collected cell lysates were incubated for 30 min on ice. After incubation, cell debris was abandoned by centrifuging at 12 000 rpm for 20 min at 4 °C. The supernatant was collected for protein concentration determination using the Bradford method (Bio‐Rad Laboratories, CA, USA). 20 µg of protein underwent SDS‐polyacrylamide gel electrophoresis and then was transferred onto a polyvinylidene fluoride membrane (PVDF) (GE, MA, USA). Bovine serum albumin (BSA) (5%) was used to block non‐specific sites on the membrane. Then the membrane was used to detect interested targets with specific antibodies.

### Co‐Immunoprecipitation (Co‐IP)

Cell lysates were harvested with IP buffer and precleared with protein A/G‐agarose beads (Santa Cruz) for 1 h at 4 °C. Anti‐PIM1, anti‐HA‐Tag, anti‐phos‐Thr/Ser, or anti‐IFNAR1 antibodies were incubated with cell lysates overnight. Protein A/G‐ agarose beads were added to collect immune complexes. The A/G‐agarose beads with immune complexes were washed with washing buffer for four times. Immune complexes were dissolved with SDS loading buffer and underwent western blot for further analysis.

### Purification of Recombinant Proteins

The expression construct of pET28a‐PIM1/pET28a‐β‐TrCP1 was transformed into Lemo21 competent (*Escherichia coli) E. coli* cells. The bacteria were cultured at 37 °C until the OD600 reached ≈0.6. Then the target protein expression in bacteria was induced by 0.8mm of isopropyl ‐β‐ D‐thiogalactopyranoside (IPTG) at 16 °C overnight. The recombinant His‐tagged PIM1 and β‐TrCP1 were purified by immobilized metal ion affinity chromatography (IMAC) using Ni‐NTA agarose (Qiagen, Germany) according to the manufacturer. A series of buffer replacements with concentration tube was used to eliminate Imidazole from the protein solution. At last concentrated proteins were stored at −80 °C for further usage.

### Immunofluorescence Assay (IF Assay)

IF assay was used to detect the localization of β‐TrCP1 and PIM1.In brief, RD cells were transfected with PIM1 and HA‐tag β‐TrCP1 plasmids for 48 h. The cells were rinsed with PBS twice and fixed with 4% paraformaldehyde for 15 min at room temperature. Then, the cells were permeabilized with 0.3% Triton X‐100 in PBS, followed by 5% BSA in PBS blocking for 2 h, and stained by incubation with anti‐PIM1 and HA‐tag antibodies at 4 °C overnight. Cells were washed with wash buffer four times, followed by incubation with Alexa Fluor 488‐conjugated anti‐mouse antibody or Alexa Fluor 594‐conjugated anti‐rabbit antibody. The nuclei were stained with DAPI for 5 min. Images were captured on a Nikon large‐field confocal system.

### Kinase Assay

PIM1 kinase assays were conducted using recombinant 6xHis‐PIM1 (WT) protein. Recombinant PIM1 protein (1 µg) and (5 µg) of recombinant β‐TrCP1 protein were incubated in 50µL of reaction buffer (50 mm Tris‐HCl (pH 7.4), 10 mm MgCl2, 0.2 mm ATP) at 30 °C for 30 min. For dephosphorylation of β‐TrCP1, Lambda protein phosphatase was added into one part of PIM1 and β‐TrCP1 mixture for another 30 min incubation. Reaction was terminated by the addition of 2 ´ SDS sample buffer (with DTT), and samples were boiled at 95 °C for 5 min, followed by phos‐tag PAGE analysis.

### Cell Membrane Fraction Assay

The cellular membrane fraction was isolated using the Membrane and Cytosol Protein Extraction Kit (Beyotime Biotechnology, P0033), following the manufacturer's protocol. Briefly, harvested cells were lysed in hypotonic buffer supplemented with protease inhibitors. After incubation on ice (15 min), homogenization was performed by 40 passes through a 26‐gauge needle. The lysate was centrifuged at 700 × g for 10 min at 4 °C to remove nuclei and unbroken cells. The supernatant was further ultracentrifuged at 14 000 × g for 30 min at 4 °C to pellet the membrane fraction. The resultant membrane pellet was solubilized in RIPA buffer for downstream analysis. Protein concentrations were quantified by BCA assay (Thermo Fisher Scientific), and membrane enrichment was validated by immunoblotting for the N‐Cadherin (membrane marker) and Hsp90 (cytosolic control).

### Flow Cytometry Assay

Cell surface expression of IFNAR1 was quantified by flow cytometry using a phycoerythrin (PE)‐conjugated anti‐human IFNAR1 antibody (R&D Systems, Catalog # FAB245P). Cells (1 × 10^6^) were washed with cold PBS containing 1% BSA (BSA in PBS). Cells were stained with the PE‐anti‐IFNAR1 antibody (10µL per test) or an isotype‐matched control PE‐IgG antibody (R&D Systems, IC003P) for 30 min at 4 °C in the dark. After washing with 1% BSA buffer, cells were resuspended in 300 µL PBS and analyzed immediately on a flow cytometer (BD Biosciences). Data from 10 000 live‐cell events (gated by FSC/SSC) were processed using FlowJo v10.8.1.

### Statistical Analysis

Quantification and statistical analysis were conducted by GraphPad Prism v9.00 (GraphPad Software Inc., USA). The results were depicted as mean ± standard deviation (SD). When comparing 2 groups, a 2‐tailed unpaired Student's t‐test was performed. A p‐value < 0.05 was regarded as statistically significant. Exact p values are reported in figures (*ns*: *p* ≥ 0.05; ^*^: *p* < 0.05; ^**^: *p* < 0.01). All experiments were performed in at least three biological replicates.

## Conflict of Interest

The authors declare no conflict of interest.

## Author Contributions

Q.W., L.Z., and C.C. contributed equally to this work and they are are co‐first authors. Q.Y.W., L.Z., and C.E.C. did experiments and collected data. Q.Y.W., Z.W.C., and M.L.H. designed the study, Q.Y.W., Z.W.C., M.Y.P., and M.L.H. analyzed the data, Q.Y.W., M.Y.P., L.Z. and M.L.H. drafted the manuscript.

## Supporting information



Supporting Information

Supporting Information

## Data Availability

The data that support the findings of this study are available from the corresponding author upon reasonable request.
