# Supplementary material for: PIM1 Attenuates Innate Immunity to Foster Coronavirus Replication through Ubiquitin Ligase β‐TrCP‐Mediated IFNAR1 Degradation
Source: Adv Sci (Weinh). 2025 Jul 6;12(37):e03487. doi: 10.1002/advs.202503487 (PMC12499414; doi:10.1002/advs.202503487)
Supplement: Supplementary file 1 — Supporting Information [file ADVS-12-e03487-s002.docx]

Supplementary Materials for

PIM1 Attenuates Innate Immunity to Foster Coronavirus Replication through Ubiquitin Ligase β-TrCP Mediated IFNAR1 Degradation

Qianya Wan^1, #^, Lin Zhu^2, #^, Cien Chen^1, #^, Li Zhong^2^, Houying Leung^1^, Wei Li^4^, Chang Xu ^4^**^,^**^5^, Xi Yao^1^, Huan Hu^1^, Mandi Wu^1^, Yuxin Hou^6^, Hin Chu^6^, Yiran Wang^1^, Sheng Chen^1^, Mingyu Pan^1,7*^, Zongwei Cai ^2*^, Ming-liang He^1,3*^

### # Those authors contributed equally.

### *Corresponding author:

Dr. Mingyu Pan

Email: mingypan@cityu.edu.hk

Dr. Zongwei Cai

Email: zwcai@hkbu.edu.hk

Dr. Ming-liang He

Professor

Department of Biomedical Sciences

City University of Hong Kong

Email: [minglihe@cityu.edu.hk](mailto:minglihe@cityu.edu.hk) or [mlhe7788@gmail.com](mailto:mlhe7788@gmail.com)

^1^Department of Biomedical Sciences and Tung Biomedical Sciences Center, City University of Hong Kong, Hong Kong, China

^2^State Key Laboratory of Environmental and Biological Analysis, Hong Kong Baptist University, Hong Kong, China.

^3^CityU Shenzhen Research Institute, Nanshan, Shenzhen, China.

^4^ Weihai Municipal Hospital, Cheeloo College of Medicine, Shandong University, Shandong, China.

^5^College of Medical Laboratory, Dalian Medical University, Dalian, Liaoning, China.

^6^State Key Laboratory of Emerging Infectious Diseases, Department of Microbiology, Li Ka Shing Faculty of Medicine, The University of Hong Kong, Hong Kong, China.

^7^School of Pharmacy, Nanjing Medical University, Nanjing, China.

**This PDF file includes:**

Supplementary Text

Figures. S1 to S5

Raw data

**
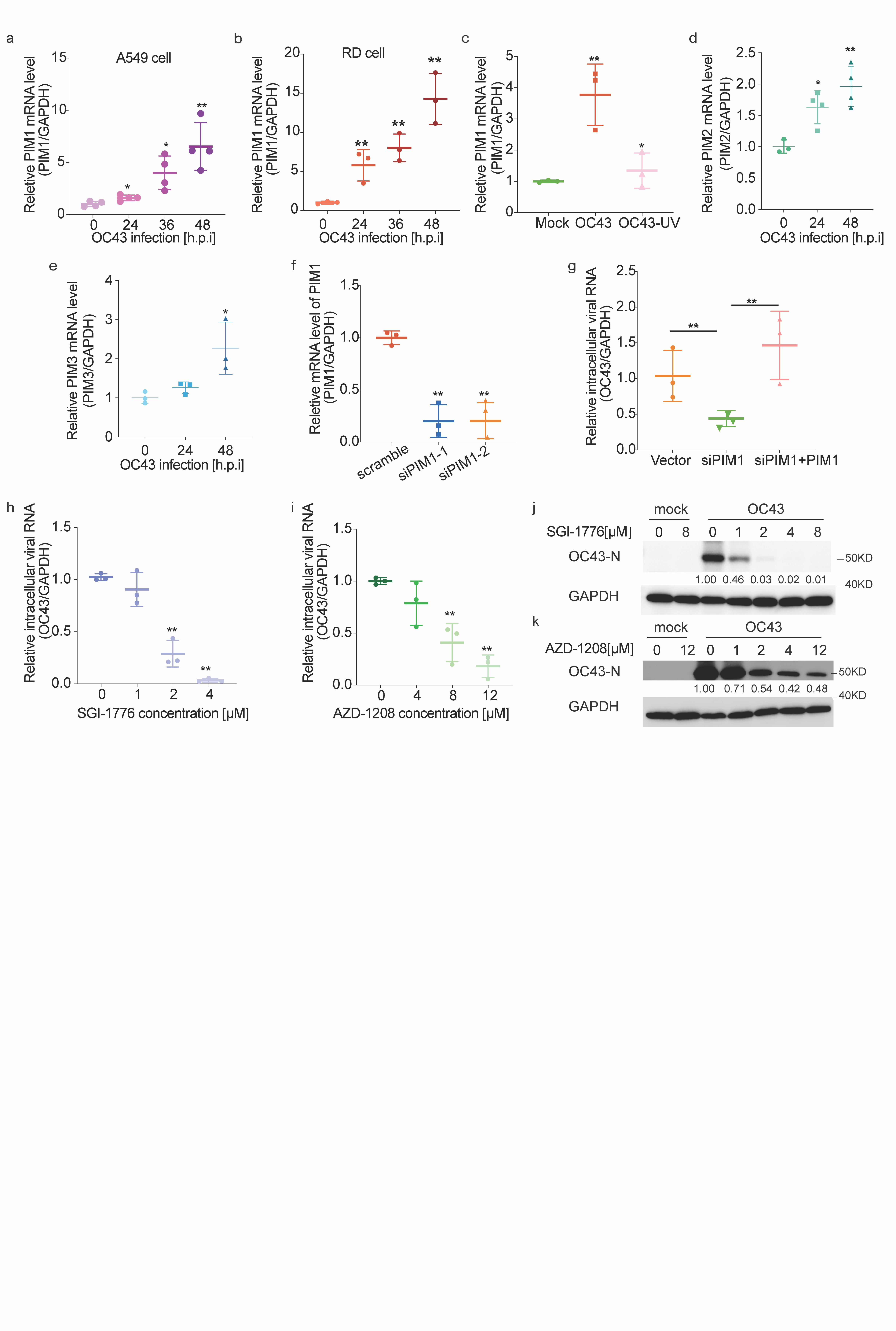
**

**Figure S1:** **PIM1 depletion or inhibition reduces OC43 and replication.** a,b) RT-qPCR analysis of PIM1 in A549 (n=4)(a) and RD (b)(n=3) cells infected with OC43 at an MOI of 1 for the indicated time points. c) Quantification of PIM1 in RD cells challenged with wild-type or UV-treated OC43 for 48h (n=3). d, e) Expression level of PIM2 and PIM3 in RD cells upon OC43 infection for 48h at an MOI of 1 (d: n=4; e: n=3). f) Verification of knocking down efficiency of PIM1 was confirmed by RT-qPCR (n=3) g) Intracellular OC43 genomic RNA level in RD cells transfected with scramble/siPIM1 siRNA and supplemented with or without PIM1 for 20 h, followed by infection with OC43 at an MOI of 0.1 for 24h.(n=3) h-k) PIM inhibitors(SGI-1776 and AZD-1208 pre-treated with RD cells for 2h, followed by the OC43 infection for 72h at the MOI of 0.01. OC43 viral RNA and N protein were determined (n=3). Statistical significance determined by Student's t-test: *p < 0.05, **p < 0.01. The densities of all proteins of interest were quantified using ImageJ and normalized to the respective control indicated beneath the bands.


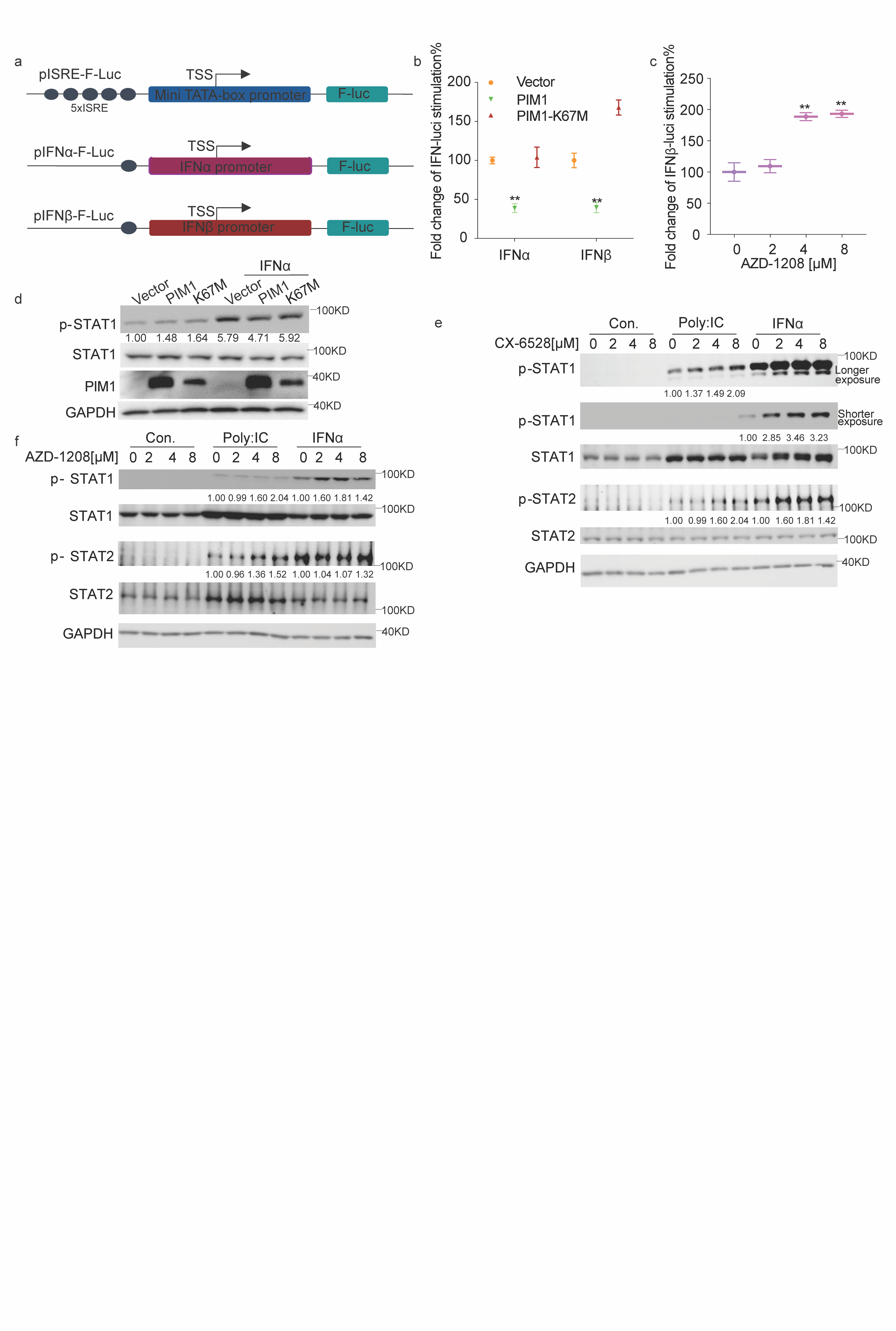


**Figure S2: Pim1 attenuated the interferon response.** a) The diagram of ISRE-luci, IFNa-luci and IFNβ-luci constructs (n=3). b) HEK293T cells were co-transfected with IFNa-luci/IFNβ-luci, pRF and vector/PIM1/PIM1 K67M plasmids for 36h. Cell lysate was collected for a dual-luciferase assay(n=3). c) Dual-luciferase assays were conducted after RD cells were pre-treated with PIM1 inhibitor AZD-1208 at the indicated concentration for 2h, followed by the transfection of IFN-luci and PRF plasmids for 36h. d) Immunoblots analysis of HEK293T cells ectopically expressed with vector/PIM1/PIM1 K67M followed by stimulated of IFNα for 30 mins. e,f) Protein level of p-STAT1/2 in HEK293T cells treated with PIM1 inhibitor CX-6528 (e) or AZD-1208 (f) for 24h followed by the stimulation with/without poly: IC for 16 h, or IFNα for 1h. Statistical significance determined by Student's t-test: *p < 0.05, **p < 0.01. The densities of all proteins of interest were quantified using ImageJ and normalized to the respective control indicated beneath the bands.


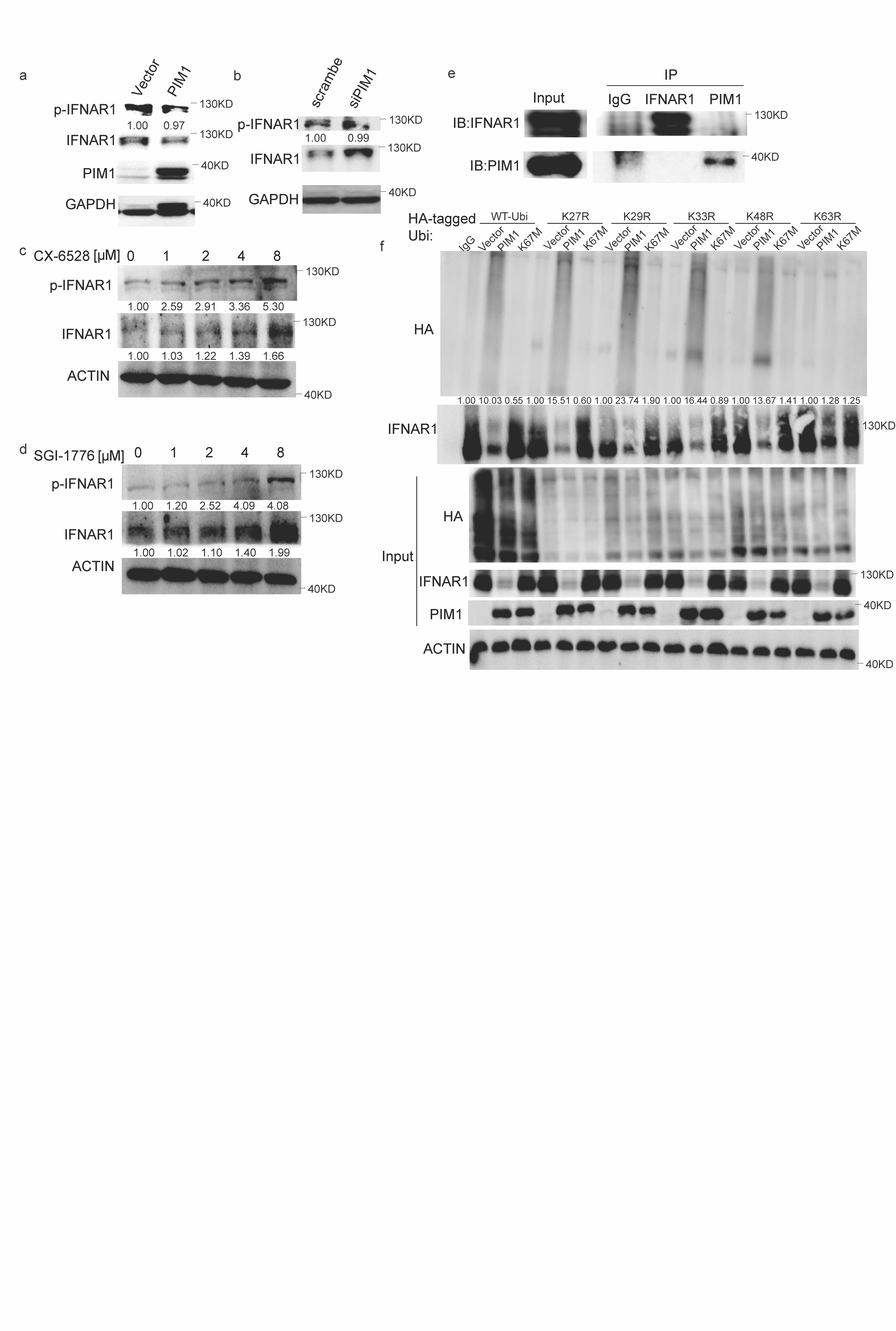


**Figure S3: PIM1 could not enhance IFNAR1 phosphorylation.** a-d) The phosphorylated level of IFNAR1 in HEK293T cells after PIM1 was ectopically expressed (a), interfered by specific siRNAs (b) for 48h, or treated with PIM inhibitors(c, d). e)Immunoprecipitation of PIM1 or IFNAR1 in HEK293T cells after co-expression of PIM1 and IFNAR1. f) Ubiquitination level of IFNAR1 with different Lysine-linked ubiquitin after co-transfection of IFNAR1 with HA-tagged WT, K27R, K29R, K33R,K48R and K63R ubiquitin constructs and WT PIM1 or K67M plasmids. The densities of all proteins of interest were quantified using ImageJ and normalized to the respective control indicated beneath the bands.

**
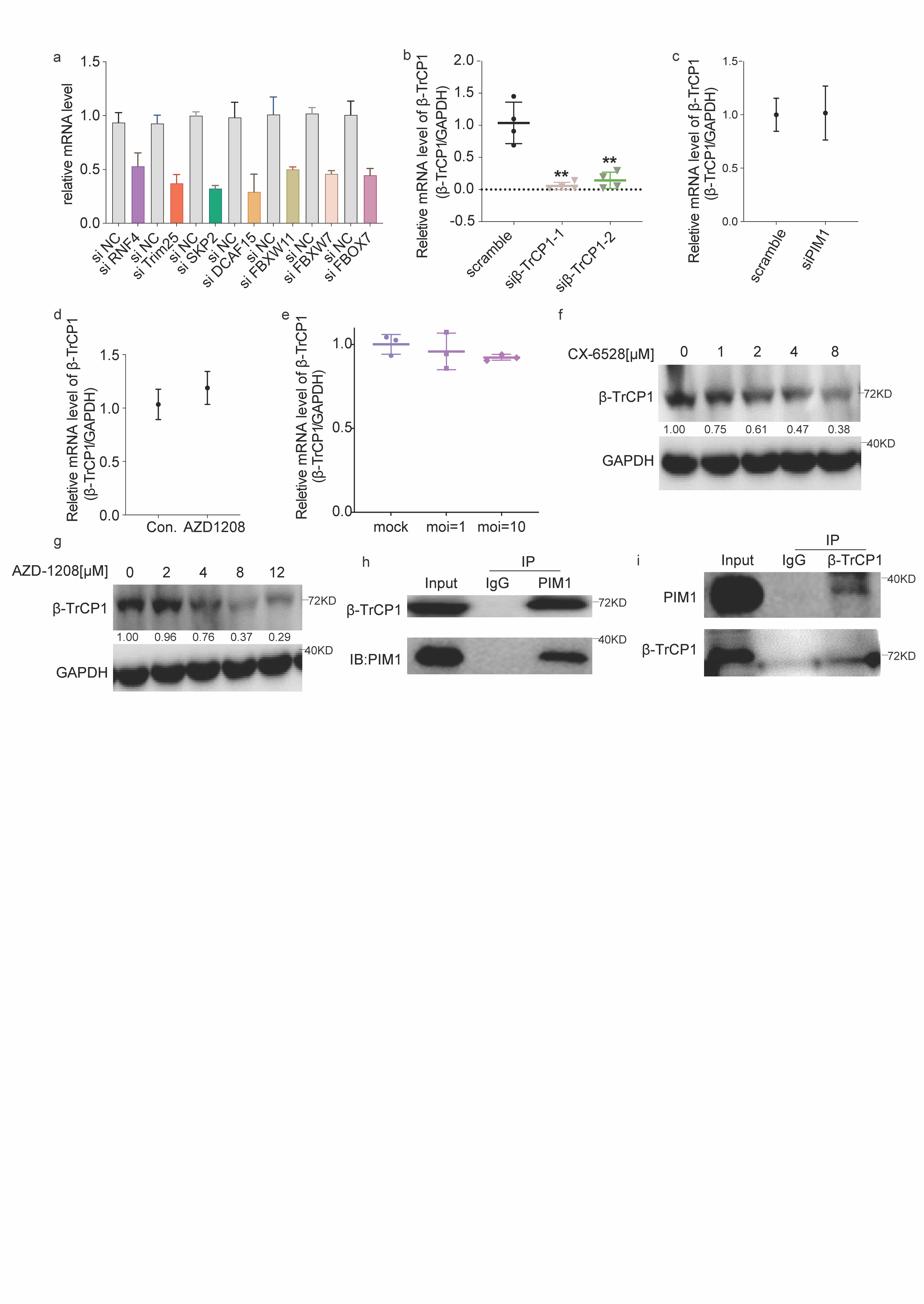
**

**Figure S4: PIM1 interacted with** β**-TrCP1 and affected its expression**. a) Knockdown efficiency of E3 ligases (n=3). b)RT-qPCR analysis of β-TrCP1 in RD cells were transfected with scramble/ β-TrCP1 specific siRNA followed by the challenge of HCoV-OC43 at an MOI of 0.1 for 24h (n=4). c-e) Quantification of β-TrCP1 in RD cells interfered with PIM1 with specific siRNA(c), 8 µM of CX-6258 (d) or HCoV-OC43 at an MOI of 1 or 10 for 36h (e) (n=3). f, g) Protein level of β-TrCP1 in HEK293T cells treated with the indicated concentration of PIM1 inhibitor CX-6258/AZD-1208 for 48h. h, i) Co-IP experiments were conducted after HEK293T cells were transfected with PIM1 and β-TrCP1 plasmids. Statistical significance determined by Student's t-test: *p < 0.05, **p < 0.01. The densities of all proteins of interest were quantified using ImageJ and normalized to the respective control indicated beneath the bands.


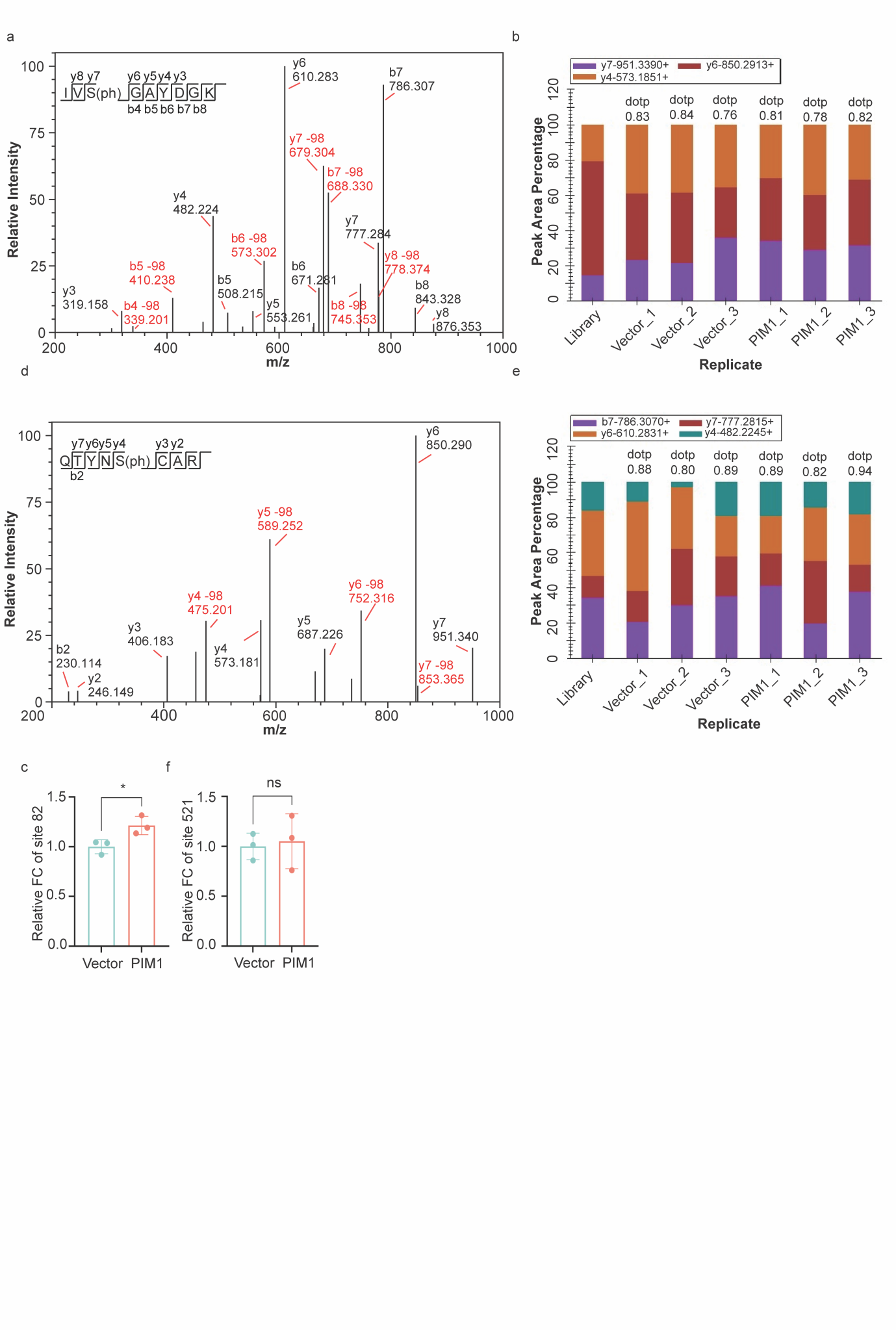


**Figure S5: Phosphorylation level of β-TrCP1 was changed after modulating PIM1.** a-f) PIM1 was overexpressed in HEK293T cells, and the β-TrCP1 was precipitated by β-TrCP1 specific antibody. Then precipitated β-TrCP1 underwent mass spectrum using site phosphorylated standard peptide (n=3). Product ion spectrums of peptides containing Ser82 (a) and Ser521 site (d). Replicate comparison of product ions containing Ser82 (b) and Ser521 site (e) Relative fold change of intercellular TrCP phosphorylation levels at Ser82 (c) and Ser521 sites (f). Statistical significance determined by Student's t-test: *p < 0.05, **p < 0.01.
