# Supplementary material for: PIM1 Attenuates Innate Immunity to Foster Coronavirus Replication through Ubiquitin Ligase β‐TrCP‐Mediated IFNAR1 Degradation
Source: Adv Sci (Weinh). 2025 Jul 6;12(37):e03487. doi: 10.1002/advs.202503487 (PMC12499414; doi:10.1002/advs.202503487)
Supplement: Supplementary file 2 — Supporting Information [file ADVS-12-e03487-s001.pdf]

ADVANCED SCIENCE
Open Access

Supporting Information

for *Adv. Sci.*, DOI 10.1002/advs.202503487

PIM1 Attenuates Innate Immunity to Foster Coronavirus Replication through Ubiquitin Ligase β-TrCP-Mediated IFNAR1 Degradation

Qianya Wan, Lin Zhu, Cien Chen, Li Zhong, Houying Leung, Wei Li, Chang Xu, Xi Yao, Huan Hu, Mandi Wu, Yuxin Hou, Hin Chu, Yiran Wang, Sheng Chen, Mingyu Pan, Zongwei Cai* and Ming-liang He**

RAW/UNPROCESSED BLOTS

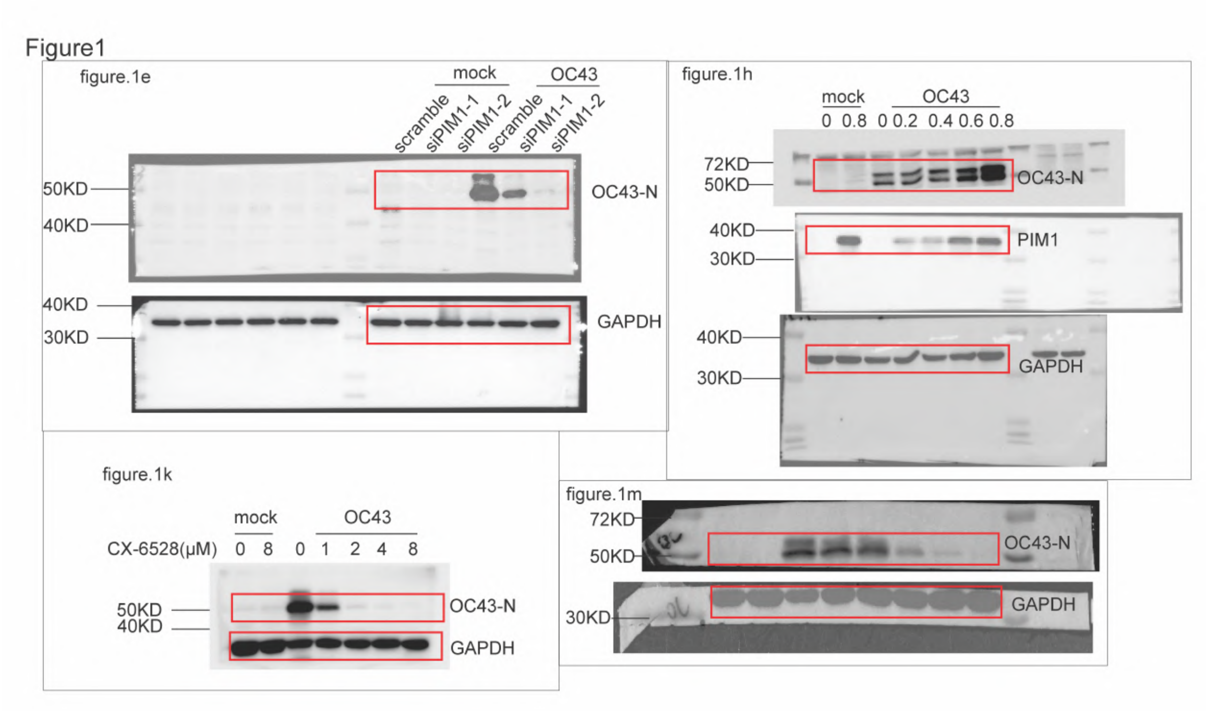

Figure2

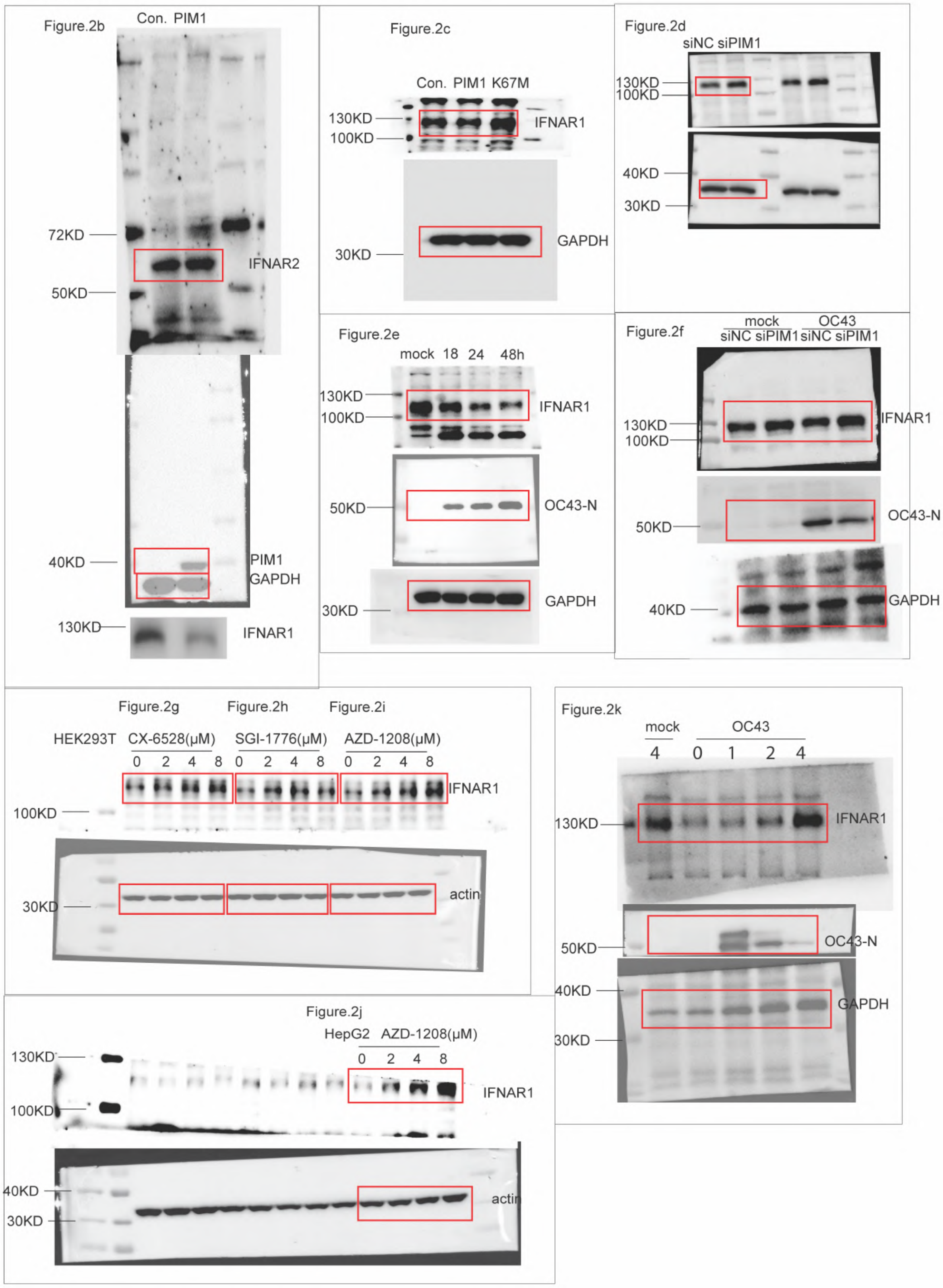
Figure.2b
Con. PIM1
72KD
IFNAR2
50KD
40KD
PIM1
GAPDH
130KD
IFNAR1
Figure.2c
Con. PIM1 K67M
130KD
100KD
IFNAR1
30KD
GAPDH
Figure.2d
siNC siPIM1
130KD
100KD
40KD
30KD
Figure.2e
mock 18 24 48h
130KD
100KD
IFNAR1
50KD
OC43-N
30KD
GAPDH
Figure.2f
mock
OC43
siNC siPIM1 siNC siPIM1
130KD
100KD
IFNAR1
50KD
OC43-N
40KD
GAPDH
Figure.2g
Figure.2h
Figure.2i
HEK293T CX-6528(μM) SGI-1776(μM) AZD-1208(μM)
0 2 4 8 0 2 4 8 0 2 4 8
IFNAR1
100KD
30KD
actin
Figure.2k
mock
OC43
4 0 1 2 4
130KD
IFNAR1
50KD
OC43-N
40KD
GAPDH
30KD
Figure.2j
HepG2 AZD-1208(μM)
0 2 4 8
130KD
100KD
IFNAR1
40KD
30KD
actin

Figure3

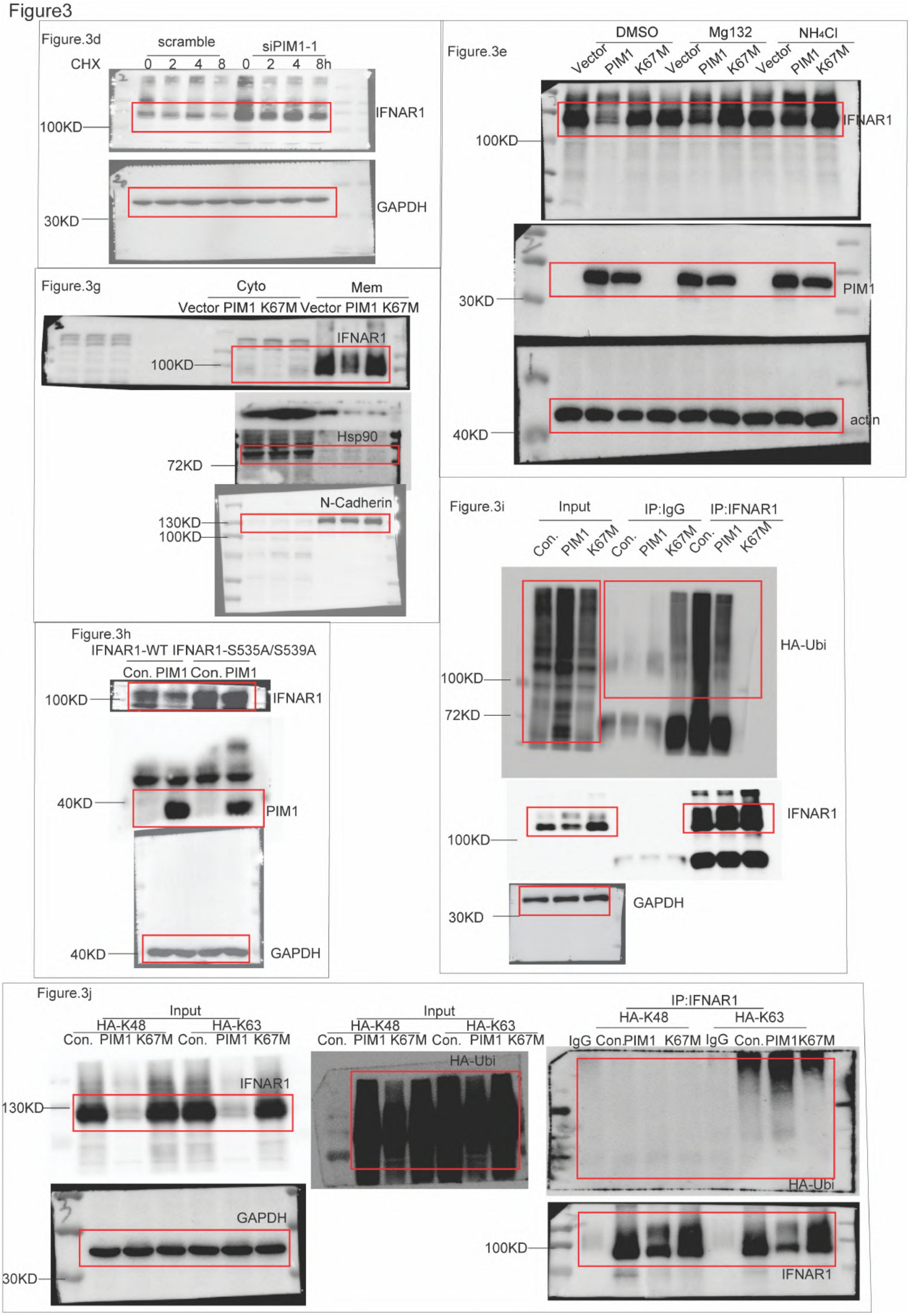
Figure.3d
scramble
siPIM1-1
CHX
0 2 4 8 0 2 4 8h
100KD
IFNAR1
30KD
GAPDH
Figure.3e
DMSO
Mg132
NH4Cl
Vector PIM1 K67M Vector PIM1 K67M Vector PIM1 K67M
100KD
IFNAR1
30KD
PIM1
40KD
actin
Figure.3g
Cyto
Mem
Vector PIM1 K67M Vector PIM1 K67M
IFNAR1
100KD
Hsp90
72KD
N-Cadherin
130KD
100KD
Figure.3h
IFNAR1-WT IFNAR1-S535A/S539A
Con. PIM1 Con. PIM1
100KD
IFNAR1
40KD
PIM1
40KD
GAPDH
Figure.3i
Input
IP:IgG
IP:IFNAR1
Con. PIM1 K67M Con. PIM1 K67M Con. PIM1 K67M
HA-Ubi
100KD
72KD
IFNAR1
100KD
GAPDH
30KD
Figure.3j
Input
HA-K48
HA-K63
Con. PIM1 K67M Con. PIM1 K67M
IFNAR1
130KD
GAPDH
30KD
Input
HA-K48
HA-K63
Con. PIM1 K67M Con. PIM1 K67M
HA-Ubi
IP:IFNAR1
HA-K48
HA-K63
IgG Con.PIM1 K67M IgG Con.PIM1K67M
HA-Ubi
100KD
IFNAR1

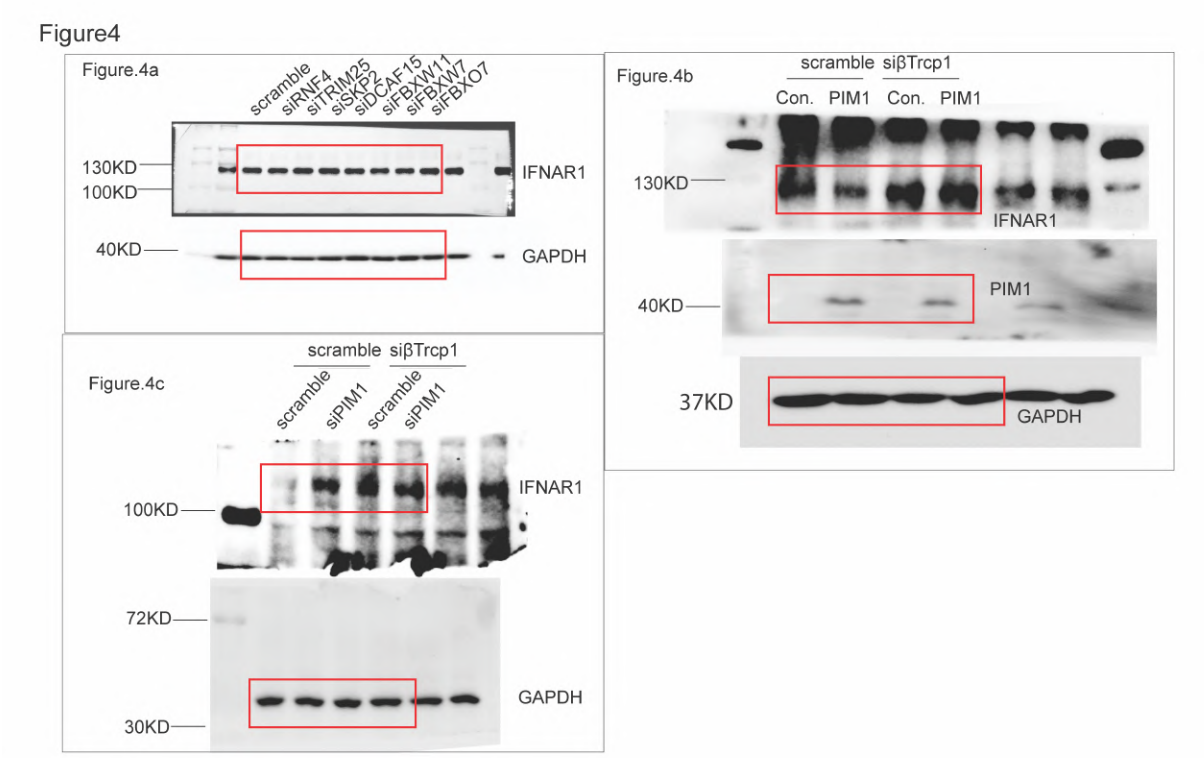
Figure4
Figure.4a
scramble
siRNF4
siTRIM25
siSKP2
siDCAF15
siFBXW11
siFBXW7
siFBXO7
130KD
100KD
IFNAR1
40KD
GAPDH
Figure.4b
scramble
siβTrcp1
Con.
PIM1
Con.
PIM1
130KD
IFNAR1
40KD
PIM1
37KD
GAPDH
Figure.4c
scramble
siβTrcp1
scramble
siPIM1
scramble
siPIM1
100KD
IFNAR1
72KD
30KD
GAPDH

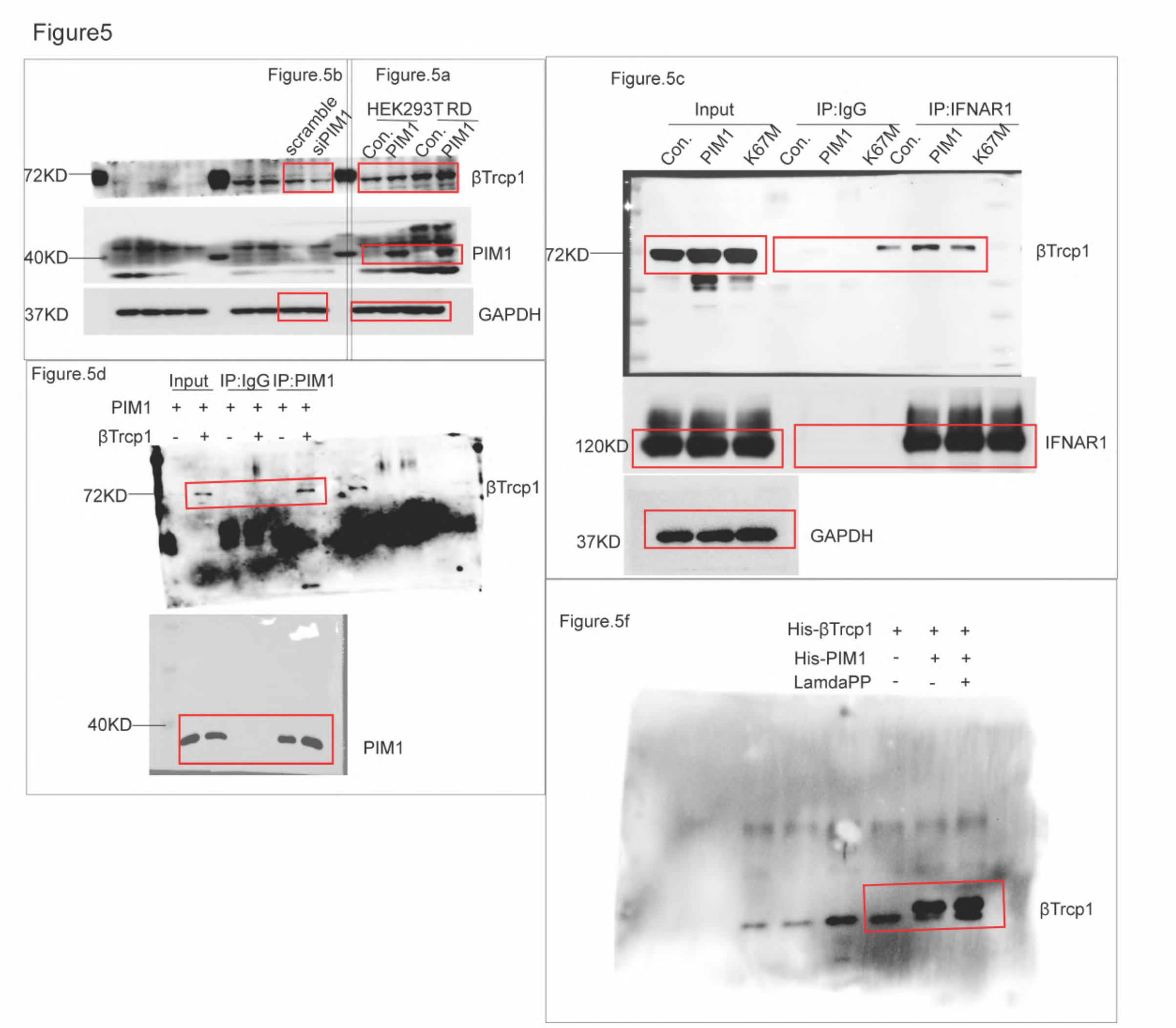
Figure5
Figure.5b
Figure.5a
scramble
siPIM1
HEK293T
RD
Con.
PIM1
Con.
PIM1
72KD
βTrcp1
40KD
PIM1
37KD
GAPDH
Figure.5c
Input
IP:IgG
IP:IFNAR1
Con.
PIM1
K67M
Con.
PIM1
K67M
Con.
PIM1
K67M
72KD
βTrcp1
120KD
IFNAR1
37KD
GAPDH
Figure.5d
Input
IP:IgG
IP:PIM1
PIM1 + + + + + +
βTrcp1 - + - + - +
72KD
βTrcp1
40KD
PIM1
Figure.5f
His-βTrcp1 + + +
His-PIM1 - + +
LamdaPP - - +
βTrcp1

Figure6

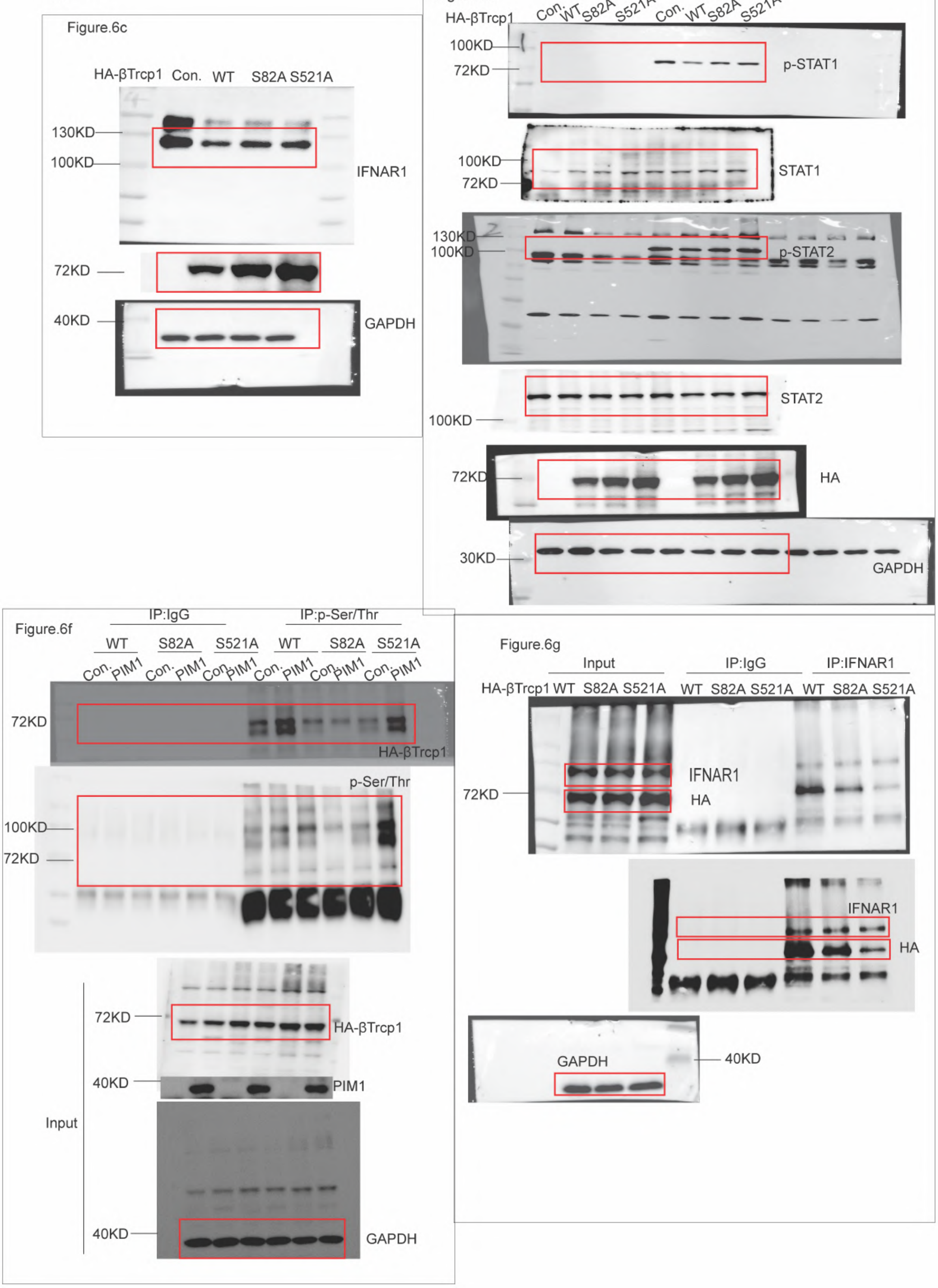

Figure.6c
HA-βTrcp1 Con. WT S82A S521A
130KD
100KD
IFNAR1
72KD
40KD
GAPDH
Figure.6d
IFNα
HA-βTrcp1 Con. WT S82A S521A Con. WT S82A S521A
100KD
72KD
p-STAT1
STAT1
130KD
p-STAT2
STAT2
HA
30KD
GAPDH
Figure.6f
IP:IgG
IP:p-Ser/Thr
WT S82A S521A WT S82A S521A
Con. PIM1 Con. PIM1 Con. PIM1 Con. PIM1 Con. PIM1 Con. PIM1
HA-βTrcp1
p-Ser/Thr
40KD
PIM1
Input
Figure.6g
Input
IP:IgG
IP:IFNAR1
HA-βTrcp1 WT S82A S521A WT S82A S521A WT S82A S521A
IFNAR1
HA

Figure S1

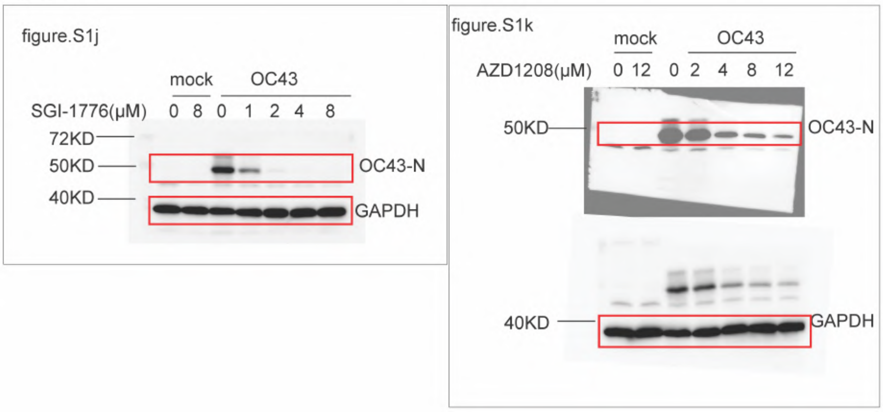
figure.S1j
mock
OC43
SGI-1776(μM)
0 8 0 1 2 4 8
72KD
50KD
40KD
OC43-N
GAPDH
figure.S1k
mock
OC43
AZD1208(μM)
0 12 0 2 4 8 12
50KD
OC43-N
40KD
GAPDH

8

Figure S2

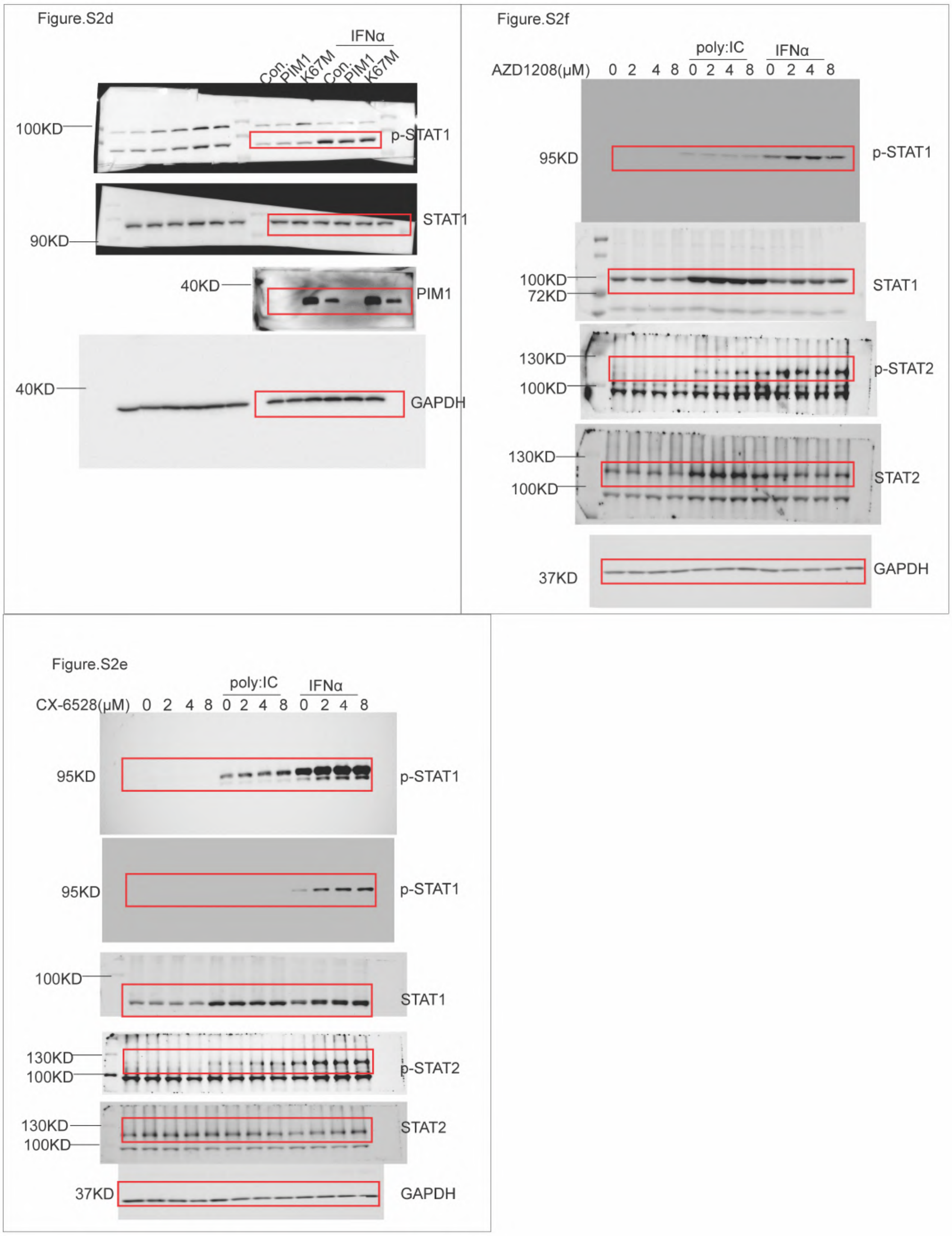
Figure.S2d
IFNα
Con.
PIM1
K67M
Con.
PIM1
K67M
100KD
p-STAT1
90KD
STAT1
40KD
PIM1
40KD
GAPDH
Figure.S2f
poly:IC
IFNα
AZD1208(μM) 0 2 4 8 0 2 4 8 0 2 4 8
95KD
p-STAT1
100KD
72KD
STAT1
130KD
100KD
p-STAT2
130KD
100KD
STAT2
37KD
GAPDH
Figure.S2e
poly:IC
IFNα
CX-6528(μM) 0 2 4 8 0 2 4 8 0 2 4 8
95KD
p-STAT1
95KD
p-STAT1
100KD
STAT1
130KD
100KD
p-STAT2
130KD
100KD
STAT2
37KD
GAPDH

Figure.S3

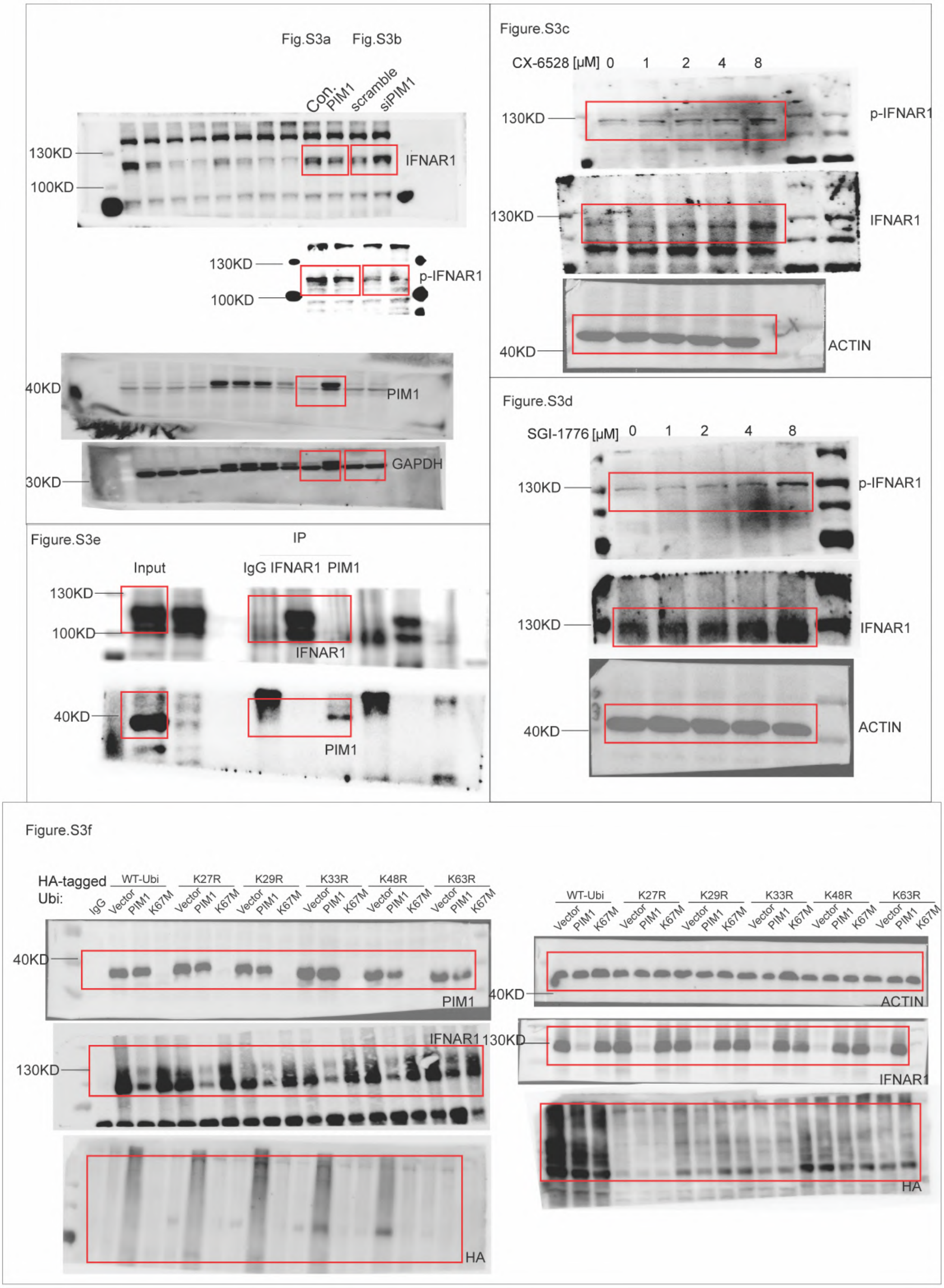

Fig.S3a
Fig.S3b
Con.
PIM1
scramble
siPIM1
130KD
100KD
IFNAR1
130KD
100KD
p-IFNAR1
40KD
PIM1
30KD
GAPDH
Figure.S3c
CX-6528 [μM] 0 1 2 4 8
130KD
p-IFNAR1
130KD
IFNAR1
40KD
ACTIN
Figure.S3d
SGI-1776 [μM] 0 1 2 4 8
130KD
p-IFNAR1
130KD
IFNAR1
40KD
ACTIN
Figure.S3e
IP
Input
IgG IFNAR1 PIM1
130KD
100KD
IFNAR1
40KD
PIM1
Figure.S3f
HA-tagged Ubi:
WT-Ubi
K27R
K29R
K33R
K48R
K63R
IgG
Vector
PIM1
K67M
40KD
PIM1
IFNAR1
130KD
HA
40KD
ACTIN
130KD
IFNAR1
HA

Figure.S4

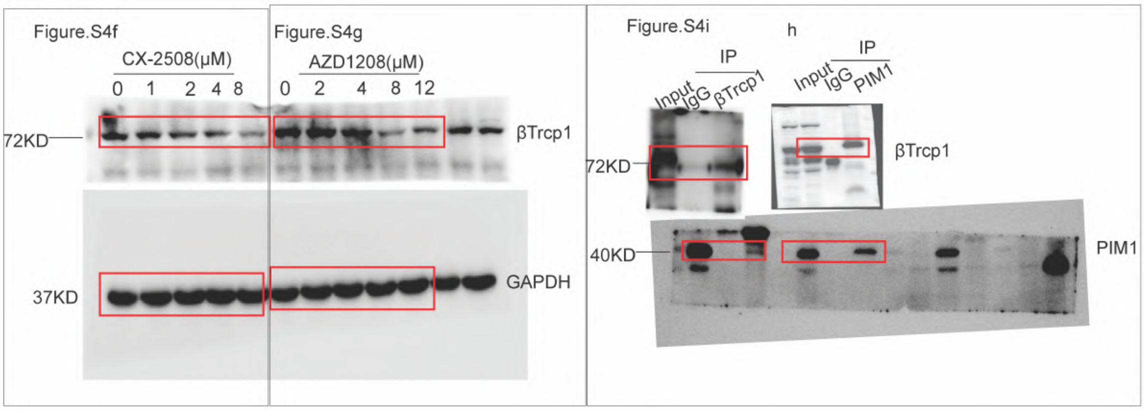

11

12
